# The Clinical Impact of Real-Time fMRI Neurofeedback on Emotion Regulation: A Systematic Review

**DOI:** 10.3390/brainsci14070700

**Published:** 2024-07-12

**Authors:** Nadja Tschentscher, Julia C. Tafelmaier, Christian F. J. Woll, Oliver Pogarell, Maximilian Maywald, Larissa Vierl, Katrin Breitenstein, Susanne Karch

**Affiliations:** 1 Section of Clinical Psychology and Psychophysiology, Department of Psychiatry and Psychotherapy, LMU University Hospital, LMU Munich, Nußbaumstr. 7, 80336 Munich, Germany; nadja.tschentscher@med.uni-muenchen.de (N.T.); julia.tafelmaier@psy.lmu.de (J.C.T.); oliver.pogarell@med.uni-muenchen.de (O.P.);; 2 Section of Clinical Psychology of Children and Adolescents, Department of Psychology and Educational Sciences, Ludwig Maximilian University of Munich, Leopoldstr. 13, 80802 Munich, Germany; christian.woll@psy.lmu.de; 3 Section of Clinical Psychology and Psychological Treatment, Department of Psychology and Educational Sciences, Ludwig Maximilian University of Munich, Leopoldstr. 13, 80802 Munich, Germany

**Keywords:** real-time fMRI, neurofeedback, emotion regulation, neuromodulation, systematic review

## Abstract

Emotion dysregulation has long been considered a key symptom in multiple psychiatric disorders. Difficulties in emotion regulation have been associated with neural dysregulation in fronto-limbic circuits. Real-time fMRI-based neurofeedback (rt-fMRI-NFB) has become increasingly popular as a potential treatment for emotional dysregulation in psychiatric disorders, as it is able to directly target the impaired neural circuits. However, the clinical impact of these rt-fMRI-NFB protocols in psychiatric populations is still largely unknown. Here we provide a comprehensive overview of primary studies from 2010 to 2023 that used rt-fMRI-NFB to target emotion regulation. We assessed 41 out of 4001 original studies for methodological quality and risk of bias and synthesised concerning the frequency of significant rt-fMRI-NFB-related effects on the neural and behaviour level. Successful modulation of brain activity was reported in between 25 and 50 percent of study samples, while neural effects in clinical samples were more diverse than in healthy samples. Interestingly, the frequency of rt-fMRI-NFB-related behavioural improvement was over 75 percent in clinical samples, while healthy samples showed behavioural improvements between 0 and 25 percent. Concerning clinical subsamples, rt-fMRI-NFB-related behavioural improvement was observed in up to 100 percent of major depressive disorder (MDD) and post-traumatic stress disorder (PTSD) samples. Substance use samples showed behavioural benefits ranging between 50 and 75 percent. Neural effects appeared to be less frequent than behavioural improvements: most neural outcomes ranged between 25 and 50 percent for MDD and substance use and between 0 and 25 percent for PTSD. Using multiple individualised regions of interest (ROIs) for rt-fMRI-NFB training resulted in more frequent behavioural benefits than rt-fMRI-NFB solely based on the amygdala or the prefrontal cortex. While a significant improvement in behavioural outcomes was reported in most clinical studies, the study protocols were heterogeneous, which limits the current evaluation of rt-fMRI-NFB as a putative treatment for emotional dysregulation.

## 1. Introduction

Emotion regulation allows us to influence what kind of emotions are occurring, the intensity of emotions, and their duration [1]. Emotion dysregulation has been considered a key factor for the development, severity, and persistence of symptoms in multiple psychiatric disorders [2], such as anxiety disorders, major depression, eating disorders, substance-related disorders, borderline personality disorder, and obsessive-compulsive disorder [3,4]. Thus, improving emotion regulation has been the focus of many cognitive-behavioural psychotherapeutic interventions, such as dialectical behavioural therapy [5], schema therapy [6], as well as psychodynamic interventions [7].

On the neural level, it has been hypothesised that neural alterations associated with dysfunctional emotion regulation might be associated with impaired top-down control of prefrontal regions over limbic structures [8,9]. The anterior insula and amygdala, for example, have repeatedly been associated with the experience of emotions irrespective of the valence of the emotions [10]. Thus, these brain areas have been assigned to the intensity of emotional experiencing rather than the valence [11]. By contrast, especially lateral prefrontal areas have been demonstrated to be involved in cognitive emotion regulation efforts [12]. In addition, neural differences between psychiatric patients and healthy controls have been reported for fMRI-based connectivity measures between frontal and limbic structures [13,14,15,16]. Moreover, dysfunctional emotion regulation has been associated with hyperactivation of the amygdala [17,18,19] and hypoactivation of prefrontal brain regions [13,20,21]. Such alterations in cortical and subcortical brain regions have been observed in patients diagnosed with obsessive-compulsive disorder [13,18,22], bipolar disorder [20,23], major depression [14,19,24], anxiety disorders [16,21], post-traumatic-stress-disorder [25,26], borderline personality disorder [17,27,28], and substance use disorders [29,30,31]. Additional brain regions associated with emotion regulation dysfunctions in psychiatric populations are the insula [32,33,34], the anterior cingulate cortex [16,35,36], the orbito-frontal cortex [22,23], and the ventral striatum [14,19,22].

To address emotion regulation dysfunction on the neural level, several techniques have been developed and explored for their capacity to modulate neuronal responses and/or to induce neuroplastic processes. Real-time fMRI neurofeedback (rt-fMRI-NFB) is a novel technique for learning the self-regulation of the brain. During rt-fMRI-NFB the blood-oxygen-level dependent (BOLD) signal of one or more brain areas is measured and presented to the subject in the MRI scanner. The subject aims to gain more control over the activity of these areas and the mental state corresponding with these brain regions by increasing or decreasing neuronal responses or influencing the connectivity between various target areas. Within neural regions related to emotion regulation, it could be shown that even one session of rt-fMRI-NFB resulted in the modification of region-specific brain activity [11,33,37] and large-scale network connectivity [38,39]. Namely, modification of neural activation levels due to rt-fMRI-NFB was demonstrated for the amygdala, the prefrontal cortex, the insula, and the anterior cingulate cortex. Evidence for some of these regions was reported for patients diagnosed with affective disorders [14,37,40], substance use disorders [41,42], borderline personality disorder [43], obsessive-compulsive disorder [44,45], and post-traumatic stress disorder [25,37,46], respectively. Thus, rt-fMRI-NFB might function as a potential therapeutic tool or as an add-on treatment that is able to directly target one of the underlying neural mechanisms of emotion dysregulation.

There has been a vastly increasing number of rt-fMRI-NFB studies over the past decade [33]. However, previous reviews on emotion regulation have primarily addressed methodological aspects of rt-fMRI-NFB [11,33,37]. Yet, there is no systematic review or meta-analysis focussing on the clinical impact of emotion regulation related rt-fMRI-NFB. However, evaluating the clinical impact is critical for the putative function of rt-fMRI-NFB as a therapeutic tool [47,48]. Moreover, no systematic assessment of study quality has been conducted by previous reviews.

In this preregistered systematic review, we assessed and quantified the clinical impact of emotion regulation related rt-fMRI-NFB on both the neural and behavioural level for a range of psychiatric disorders. A comprehensive assessment of study quality was performed for all included primary studies. We have synthesised and quantified the impact of rt-fMRI-NFB on neural activation levels based on specific brain regions of interest, as well as based on whole-brain measures. Namely, we predicted a high frequency of significant rt-fMRI-NFB–dependent changes in cortico-subcortical circuits across original studies. Those highly frequent significant effects may be reported by original studies for, e.g., the prefrontal cortex, the anterior cingulate cortex, the insula, the amygdala, and the striatum. We also hypothesised a high frequency of rt-fMRI-NFB–induced improvement in behavioural outcome measures related to emotion regulation across original studies. Although there was no a priori exclusion of study groups in our search strategy, our synthesis of data focussed on psychiatric disorders for which sufficient primary rt-fMRI-NFB evidence exists including major depressive disorder (MDD), substance use disorders, and post-traumatic stress disorder (PTSD). The current review focuses on rt-fMRI-NFB and does not include studies that included other NFB strategies and/or the comparison of different NFB techniques.

## 2. Methods

### 2.1. Pre-Registration

To ensure transparency and reproducibility of our work, this project was preregistered with the Open Science Framework (Date of Preregistration: 3 May 2023; see https://osf.io/dwjem, accessed on 21 May 2023) adhering to PRISMA 2020 guidelines [49]; see Supplementary Table S1 for the PRISMA Checklist of this review. No registration at PRISMA 2020 was performed. The search strategy, literature hits, excluded search hits, and detailed study quality and risk of bias assessment were uploaded in common file formats (see https://osf.io/9ud5q/?view_only=694fd5cccb8e4a67b6acfc4b727c8f03, assessed on 12 June 2024). No changes to the preregistration protocol were necessary.

### 2.2. Inclusion Criteria

We included primary studies published in English by international, peer-reviewed journals. Only studies describing human subjects over the age of 18 were included. Studies investigating either healthy populations or those diagnosed with a psychiatric disorder according to international diagnostic manuals (e.g., ICD-10, DSM-IV, DMS-V) were included. Except in samples with nicotine use, participants were selected based on cigarette consumption rather than any formal diagnosis. We excluded review articles, meta-analyses, non-primary studies, and studies with no human subjects, such as animal, genetic, or computational studies. All included studies used rt-fMRI-NF to modulate emotion regulation-related brain circuits and/or behaviour. We excluded studies using other neuroscientific methods or paradigms, such as EEG-neurofeedback or combined EEG-fMRI neurofeedback. Studies investigating rt-fMRI-NFB for any non-emotion-regulation-related concepts and/or regions of interest were excluded as well. Since our hypotheses concern basic affect regulation, we excluded studies investigating complex emotional phenomena, such as pain, self-blame, or tenderness/anguish. We included craving within the context of substance use samples, though, as this is considered an important clinical indicator of symptom severity [50] and a common rt-fMRI-NFB measure in this clinical population. Only studies published from January 2010 onwards were included to ensure comparable methodological quality of rt-fMRI-NFB paradigms.

### 2.3. Data Sources and Search Terms

The following online databases were included in the literature search: the Cochrane Library, Embase, PsychInfo, PubMed, Scopus, and Web of Science. We based our search term on a recent systematic review providing an overview of methodological aspects of rt-fMRI-NFB studies for emotion regulation processes [11]: (fMRI OR functional magnetic resonance imag* OR functional MRI OR functional imag*) AND (neurofeedback OR neuro feedback OR neuro-feedback OR feedback) AND (emot* OR affect*).

The same search term was applied to all data bases, “publication year” was set to 2010 and onwards, and “language” was limited to “English”. For PsychInfo, “linked full texts” was deactivated, “publication type” set to “peer-reviewed journal”, and “document type” set to “journal article”. For Web of Science, “type” was limited to “article” and “early access”. For The Cochrane Library, only results in the tab “trials” were considered further. For Scopus, “document type” was set to ”article” and “source type” to “journal”. There were no other deviations from the default settings of the databases. Additionally, a manual search of reference lists in relevant articles and reviews was performed.

### 2.4. Study Selection and Data Extraction

**Figure 1:**
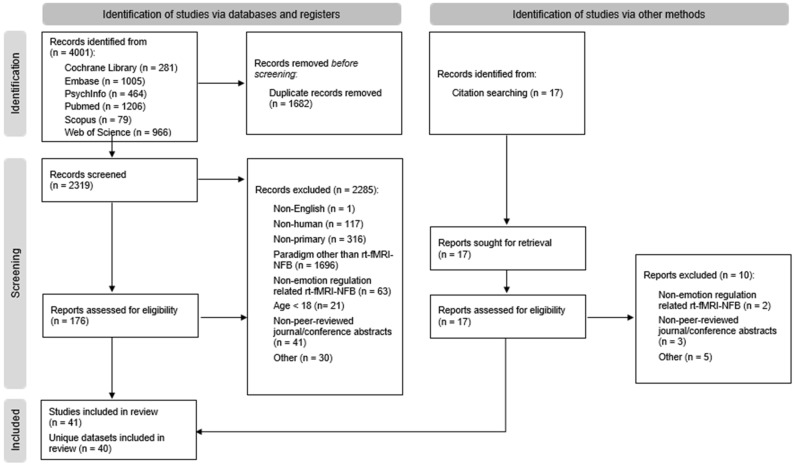
PRISMA 2020 [52] flow diagram outlining the data selection process.

The study selection and data extraction were carried out according to the PRISMA-guidelines [49] and performed by two members of the research team (J.T., N.T.). Literature hits of all databases were exported into Endnote [51] in RIS format. After duplicates were removed and titles and abstracts of all studies were screened for eligibility, followed by a full-text screening for those studies that met our eligibility criteria during the title and abstract screening. Using the described search settings, we received a total of 4001 hits (see Figure 1 for the flow diagram): 281 hits for The Cochrane Library, 1005 hits for Embase, 464 hits for PsychInfo, 1206 hits for Pubmed, 79 for Scopus, and 966 for Web of Science. After the exclusion of ineligible studies, 41 studies were included in this systematic review. All excluded studies were listed together with their respective reason for exclusion within one Excel-file accessible via the OSF (see osf.io/9ud5q, assessed on 21 May 2023) (see Figure 1 for exclusion reasons). The first authors (N.T. and J.C.T.) cross-checked the excluded and included studies.

From the included studies, the following data were extracted: study citation, author(s), publication year, sample size, population type (healthy or clinical sample), mean age, and gender distribution. For clinical samples, details on psychiatric diagnoses, medication status, comorbidities, and current treatments (e.g., pharmacological, psychotherapy) were extracted as well. From the rt-fMRI-NFB protocol, the direction of modulation (up- and/or down-modulation), type of stimuli, the number of rt-fMRI-NFB training sessions and rt-fMRI-NFB runs per session, whether practice and/or transfer runs were employed, the given instructions, and the utilised mental strategies were extracted. Additionally, the type of experimental groups, the experimental conditions, and the trained brain region of interest (ROI) were extracted. Concerning the dependent variables, behavioural outcome measures, neural measures based on the ROI and whole-brain analyses, and brain–behavioural correlation measures were extracted.

### 2.5. Risk of Bias Assessment

All included studies were evaluated for study quality and risk of bias by the two first authors (N.T. and J.T.), using the Effective Public Health Practice Project’s (EPHPP) “Quality Assessment Tool for Quantitative Studies” [53]. The domains A–F (i.e., “selection bias”, “study design”, “confounders”, “blinding”, “data collection methods”, and “withdrawal and drop-outs”) were applied. Each domain was rated “1”, “2” or “3”, corresponding to study quality ratings of “strong”, “moderate” and “weak”. If no domain was scored as “weak”, the study received a global rating of “strong”. Studies were categorised as “moderate” if they received a maximum of one “weak” rating. A global rating of “weak” was given to studies with two or more “weak” ratings across domains. The following specifications were made for the quality assessment tool to meet design aspects of rt-fMRI-NFB protocols: regarding “study design”, studies employing different experimental groups, such as a clinical and non-clinical sample or an experimental and a sham control group, were considered “controlled clinical trials” and consequently given a rating of “strong”. Regarding “confounders”, one-sample studies were rated as “strong” concerning putative differences in study samples. Concerning “blinding”, we also considered the reported blinding of participants towards their group affiliation as valid, while the evaluation tool focussed on blinding of the research question only. Regarding “collection methods”, neural outcomes, such as BOLD signal change, were considered valid and reliable and thus rated as “strong”.

### 2.6. Synthesis of Study Outcomes

We categorised *condition effects*, *training effects*, *group effects*, and *transfer effects* as either “significant” or “non-significant” in brain signal measures of ROI and whole-brain analyses. The *condition effect* refers to the within-group contrast between the experimental condition, in which participants were instructed to modulate their brain activity with the help of rt-fMRI-NFB, and the control condition without active rt-fMRI-NFB-based regulation. The *training effect* refers to the within-group effect of time (e.g., the first versus last session of rt-fMRI-NFB). The *group effect* refers to the contrast between the experimental group and the control group. The *transfer effect* refers to an additional run in which the persistence of rt-fMRI-NFB–related neural modulations was assessed without applying rt-fMRI-NFB. Only corrected results were considered if both corrected and uncorrected results were reported for any of the above-mentioned effects.

In step 1, the frequencies of significant results were calculated for each outcome by dividing the number of significant results by the number of theoretically available results based on the study design. This procedure was done if at least three outcomes were available for the respective contrast across studies. In step 2, those frequencies were sorted in percentile quantiles for each effect, using steps of 25 percent (i.e., 0–25 percent, 26–50 percent, 51–75 percent, and 75–100 percent; cf. [54]). If one study reported multiple neural results due to multiple paradigms or reporting of neural effects for healthy control groups, each of those was included in quantile calculations, thus resulting in more outcome measures available than the number of included studies. *Behavioural effects* and *brain–behavioural-associations* were considered significant if there was a significant pre-post improvement in the experimental group for at least one behavioural outcome measure or a significant correlation between at least one behavioural outcome measure and an rt-fMRI-NFB related neural effect, respectively. Thus, the number of maximally available datapoints for these contrasts equals the number of studies. For a minimum of at least three outcomes across studies, the frequency of significant effects was calculated for all studies, for healthy and clinical populations, and for different clinical subgroups, respectively.

## 3. Results

### 3.1. Included Studies

A total of 41 studies were included in this systematic review (see Figure 1, Table 1). Out of those, 17 studies investigated healthy samples, 17 clinical samples, and 7 included both clinical and healthy samples. Amongst clinical studies, six described participants with MDD, six with PTSD, and nine with substance use (six studies concerning tobacco, two concerning alcohol, and one concerning cocaine). We excluded other clinical conditions, such as borderline personality disorder (N = 2 studies) and phobia (N = 1 study), due to a lack of sufficient numbers of comparable studies. The demographic data of all included studies are presented in Supplementary Table S2.

The brain regions used for the rt-fMRI-NFB protocol varied across studies: 34 percent of studies (N = 14) focussed on the amygdala, while others focussed on the prefrontal cortex (N = 4), the anterior insula (N = 2), the anterior cingulate cortex (N = 5), posterior cingulate cortex (N = 2), mesolimbic areas (N = 2), the orbitofrontal cortex (N = 1), the hippocampus (N = 1), or a combination of the previously listed regions (N = 10). Nineteen studies instructed their participants to upregulate activity, sixteen investigated downregulation, and six studies attempted both (for details, see Table 2). Thirty studies had at least one control group, of which sixteen studies employed a sham control group, in which participants received feedback that did not correspond to their current brain activity. Nine studies employed control groups without any neurofeedback, and seven studies employed healthy subjects as controls that attended the same experimental protocol as a clinical group. Practice runs (equivalent to a transfer run but employed before the training) and transfer runs were employed by 14 and 21 studies, respectively. The number of rt-fMRI-NFB sessions ranged between one and four. The number of runs per session ranged from one to six.

### 3.2. Outcomes of the Risk of Bias Assessment

The majority of studies (N = 25, 61 percent) received a global rating of “moderate” in the “Quality Assessment Tool for Qualitative Studies” [53] (see Table 1). Eleven studies received a global rating of “weak”, and five studies were rated as “strong”. Ratings of “moderate” in overall well-designed studies were mainly due to a weak rating in the domain “selection bias” (i.e., whether participants of each study were likely to represent the target population). Study designs varied between “clinical trial” and “cohort analysis”, resulting in domain ratings of “strong” and “moderate”, respectively. The domain “confounds” was rated as “strong” in most studies (N = 28, 68 percent). For the domain “blinding”, most studies (N = 33, 81 percent) received ratings of “moderate”. Strong ratings were given to most studies in the domains “data collection method” (N = 34, 82 percent) and “withdrawals and dropouts” (N = 34, 82 percent). Detailed records for our evaluation of each included study are available on the OSF.

### 3.3. Synthesis of Results from the Trained ROIs

An overview of significant effects from ROI analyses for all contrasts of interest (i.e., condition, training, group, and transfer effects), as reported by the original studies, is presented in Table 2. Overall, 56 percent of those studies delivered data on the *condition effect*, 72 percent on the *training effect*, 69 percent on the *group effect*, 28 percent on the *transfer effect*, 59 percent on *behavioural effects*, and 36 percent reported *brain-behaviour associations*.

Table 3 presents the frequency of significant outcomes, as reported by original studies, from ROI analyses based on all samples, healthy samples, and clinical samples, respectively. Across all samples, the frequency of significant neural outcomes ranged between 25 and 50 percent for effects of condition, training, group, transfer, and brain-behaviour association. The behavioural effect of the rt-fMRI-NFB training appeared to be more frequently significant across studies, falling into the 50–75 percent quantile. For healthy samples, the frequency of significant results for the effects of condition, group, transfer, and brain–behavioural association ranged between 25 and 50 percent. The effect of training appeared to be more frequent, falling into the 50–75 percent quantile, while for healthy samples, the behavioural effect of the rt-fMRI-NFB training was observed to be less frequent, falling into the 0–25 percent quantile. In contrast to healthy samples, the behavioural effect for clinical samples ranged between 75 and 100 percent, followed by the effect of condition between 50 and 75 percent, and the effect of training between 25 and 50 percent. Interestingly, though, effects of group, transfer, and brain–behaviour association appeared to be rather infrequent for clinical samples, ranging between 0 and 25 percent.

For ROI-based analyses, we also calculated the frequency of significant effects for clinical subgroups with enough study samples (i.e., MDD, PTSD, and substance use; Table 4). For MDD and PTSD patients, a behavioural effect of the rt-fMRI-NFB training ranged between 75 and 100 percent. For substance use samples, the behavioural effect was less frequent, ranging between 50 and 75 percent. The frequency of neural effects appeared to be rather heterogeneous across clinical subsamples of MDD, PTSD, and substance use. For MDD patients, the effect of condition ranged between 50 and 75 percent, while the effects of training, group, and brain–behavioural association ranged between 25 and 50 percent. The transfer effect appeared to be rather infrequent in the MDD sample, between 0 and 25 percent. For PTSD patients, most neural effects were rather infrequent, i.e., the *training effect*, *transfer effect*, and *brain–behavioural association* ranged between 0 and 25 percent. The *condition effect* was reported to be significant between 50 and 75 percent, while no evidence was found for the *group effect* at all. For the substance use samples, *condition effect* and *transfer effect* ranged between 25 and 50 percent, while *training effect*, *group effect*, and *brain–behavioural association* ranged between 0 and 25 percent.

### 3.4. Synthesis of ROI Results from Different rt-fMRI-NFB Protocols

The frequency of significant outcomes from different rt-fMRI-NFB training ROIs is presented in Table 5. A differentiation of healthy samples and clinical (sub)samples was not possible due to the lack of sufficient data for individual ROIs. More frequent behavioural benefits of the rt-fMRI-NFB training were observed for protocols using an individualised localiser approach involving multiple regions. Behavioural benefits ranging between 50 and 75 percent were specifically found in protocols including ACC, anterior insula, hippocampus, orbitofrontal cortex, PCC, and regions of the mesolimbic system. While significant neural outcomes appeared to be most frequent for rt-fMRI-NFB trainings based on the amygdala, the behavioural effect of the amygdala training appeared to be rather infrequent, ranging between 0 and 25 percent. The behavioural benefit of rt-fMRI-NFB training protocols using the PFC could not be evaluated due to the lack of sufficient data. On the neural level, effects of the PFC-training appeared to be rather infrequent, compared to protocols using the amygdala, multiple regions, or other ROIs (i.e., the ACC, anterior insula, hippocampus, orbitofrontal cortex, PCC, and regions of the mesolimbic system).

### 3.5. Synthesis of Results from Whole-Brain Analyses

For studies that reported outcomes from whole-brain analyses, a summary of significant effects for contrasts of interest is presented in Table 6. Overall, 64 percent of studies delivered data on the condition effect, 52 percent on the training effect, 52 percent on the group effect, 20 percent on the transfer effect, and 4 percent reported brain–behaviour associations. 

The frequency of significant outcomes from whole-brain analyses is presented in Table 7 for all samples, healthy samples, and clinical samples, respectively. Across all samples, the *condition effect* appeared to be most frequent in whole-brain analyses, falling into the 50-to-75 percent quantile. The frequency of the *training effect* and *group effect* ranged between 25 and 50 percent, while the *transfer effect* was less frequent (i.e., between 0 and 25 percent). For healthy samples, the *condition effect* was most frequent in whole-brain analyses too (i.e., between 50 to 75 percent), while the *training effect* and *transfer effect* ranged between 25 and 50, and the *group effect* ranged between 0 and 25 percent. For clinical samples, the *group effect* was most frequent, falling into the 50 to 75 percent quantile. *Condition effects* and *training effects* ranged between 25 and 50 percent, while the *transfer effect* appeared between 0 and 25 percent. Due to the lack of sufficient study samples, no frequency-analysis of clinical subgroups was possible for whole-brain analyses.

## 4. Discussion

In this systematic review, we evaluated the clinical impact of rt-fMRI-NFB on emotion regulation in multiple psychiatric populations (i.e., MDD, PTSD, and substance use). We assessed 41 out of 4001 original studies for methodological quality and risk of bias and synthesised the frequency of significant rt-fMRI-NFB–related effects on the neural and behaviour level.

First, our analyses showed highly frequent behavioural benefits of rt-fMRI-NFB on emotion regulation abilities in specific clinical samples. Across studies including MDD and PTSD patients, over 75 percent in behavioural improvement related to rt-fMRI-NFB was reported, while the frequency of behavioural benefits for substance use ranged between 50 and 75 percent. In contrast, healthy samples showed rather infrequent behavioural improvements between 0 and 25 percent. The latter could reflect a ceiling effect in the respective group concerning their emotion regulation abilities.

Second, neural effects of the rt-fMRI-NFB training are diverse in clinical samples and less frequent than behavioural benefits: most neural effects ranged between 25 and 50 percent for MDD and substance use and between 0 and 50 percent for PTSD. Neural effects ranged between 25 and 75 percent in healthy samples.

Third, we provide a systematic review of the targeted brain regions of rt-fMRI-NFB protocols: overall, rt-fMRI-NFB protocols that used multiple individualised training regions showed more frequent behavioural benefits (i.e., up to 75 percent) than protocols specifically focussing on the (predefined) amygdala, with behavioural effects reported up to 25 percent. Conversely, amygdala-based rt-fMRI-NFB resulted in more frequent neural effects, followed by protocols using other ROIs, such as ACC, anterior insula, hippocampus, orbitofrontal cortex, PCC, and regions of the mesolimbic system, as well as a mixture of ROIs. Out of all assessed protocols, the PFC-based rt-fMRI-NFB revealed most infrequent neural effects and no behavioural effects. Weak outcomes for PFC-based rt-fMRI-NFB have been previously reported by, for example, Linhartová, Látalová, Kóša, Kašpárek, Schmahl and Paret [11] in their review.

### 4.1. Benefit of rt-fMRI-NFB on Emotion Regulation Depends on the Clinical Condition

Our finding of more frequent behavioural effects in certain clinical populations (i.e., MDD and PTSD) in contrast to substance use is in line with evidence from previous reviews. For example, Linhartová, Látalová, Kóša, Kašpárek, Schmahl and Paret [11] reported evidence for behavioural/clinical effects of rt-fMRI-NFB in depression and PTSD, while no evidence was found for other psychiatric disorders. However, unlike our review, they did not systematically compare the frequency of rt-fMRI-NFB–induced effects across clinical groups and targeted brain regions. Evidence for clinical effects of rt-fMRI-NFB in depression and PTSD was also reported by Barreiros, Almeida, Baía and Castelo-Branco [37].

On the single-study level, Young et al. [40,89] reported the most significant neural and behavioural effects across assessed contrasts for MDD. Both studies focussed on the upregulation of multiple regions (amygdala and the horizontal segment of the intraparietal sulcus) and used sham-neurofeedback on other brain regions as control conditions. For PTSD, rt-fMRI-NFB targeting the PCC and PFC showed overall most frequent significant effects across all assessed contrasts [82,93].

### 4.2. rt-fMRI-NFB Related Neural versus Behavioural Effects

This systematic review for the first time provides evidence from data showing that behavioural/clinical effects of rt-fMRI-NFB interventions on emotion regulation are relatively frequent across clinical populations and, in some cases, more frequently reported than corresponding neural effects.

However, from our data, we cannot conclude that rt-fMRI-NFB is genuinely less effective on the neural than on the behavioural level. Only 12 out of 41 studies directly assessed the association between neural and behavioural effects in our systematic review, and effect sizes have been barely reported for behavioural effects. Also, due to lack of sufficient evidence on other neuroimaging analysis methods, we focussed on univariate ROI- and whole-brain analyses. Thus, it might be the case, especially for studies targeting multiple ROIs during rt-fMRI-NFB training, that neural effects emerge in brain regions outside predefined ROIs. For example, in this systematic review, a condition effect has been frequently reported in whole-brain analyses speaking to the existence of rt-fMRI-NFB–related neural effects outside of predefined ROIs. Moreover, neural effects might be more likely captured by other imaging measures, such as functional connectivity and resting state connectivity. For example, Zhao et al. [94] applied rt-fMRI-NFB on amygdala-prefrontal pathways and could show the effects of the training in these regions, as well as a significant reduction in anxiety in patients suffering from anxiety disorders. Morgenroth et al. [95] reported rt-fMRI-NFB–related changes in functional connectivity of a dorsolateral prefrontal-anterior cingulate cortex network, alongside with changes in trait anxiety measures. Targeting MDD via a mindfulness-based approach, Zhang, et al. [96] could show in a pilot-study both changes in the default mode network after a mindfulness-based rt-fMRI-NFB training, as well as changes in levels of mindfulness in the respective group of patients. Hence, preliminary evidence from these studies on network-based rt-fMRI-NFB might suggest that those measures, other than those from univariate fMRI analyses, could be a promising tool for providing a stronger link between neural and behavioural function in rt-fMRI-NFB based studies on psychiatric disorders.

### 4.3. Individualised Multi-Region Protocols Outperform Single-Region Protocols

Our systematic review suggests that across studies, behavioural benefits are more frequently reported in individualised multi-region rt-fMRI-NFB protocols than in those using a predefined single-region rt-fMRI-NFB approach. The respective individualised multi-region training protocols targeted a mixture of regions, including the ACC, anterior insular, hippocampus, orbitofrontal cortex, PCC, and regions of the mesolimbic system. However, predefined single-region amygdala-based rt-fMRI-NFB showed the most frequent neural effects across studies. This is a puzzling finding and perhaps suggests that while the amygdala can be efficiently trained via rt-fMRI-NFB, its link to specific psychiatric symptoms, such as depression, is less straightforward. However, while our systematic review is informative concerning effective rt-fMRI-NFB approaches for specific psychiatric conditions, the underlying study protocols were heterogenous: for example, for ROI-based rt-fMRI-NFB in patients with MDD, five studies used sham- or no-NFB conditions as control, while one study used a healthy control group. All but one of those studies used a multi-region training protocol. For PTSD, two studies used sham or no control, while four studies used healthy control groups, and all but one of those studies used a single-region training protocol. This limits the interpretability of results concerning the question of which study protocol might be most effective for a particular psychiatric disorder. Unfortunately, the significant variation in study protocols also limits the overall ability to draw definitive conclusions about the effectiveness of rt-fMRI-NFB.

## 5. Conclusions

This systematic review provides the first comprehensive overview of the frequency of neural and clinical effects in rt-fMRI-NFB studies on emotion regulation. Our data suggest that rt-fMRI-NFB is effective in reducing disorder-related symptoms in MDD and PTSD patients, while clinical outcomes are less frequent in patients diagnosed with substance use disorders. The link between neural and behavioural effects is less strong overall, as neural effects from univariate fMRI analyses appeared to be less frequent than respective clinical outcomes across all assessed clinical conditions, and only a few studies directly assessed brain-behaviour associations. Based on our data, we suggest (i) using rt-fMRI-NFB training protocols with individualised, localiser-based regions in contrast to protocols with a predefined region; (ii) considering other than univariate fMRI measures, such as functional and resting state connectivity; and (iii) systematically assessing effects of the training on the neural level by means of brain-behaviour correlations.

## Figures and Tables

**Table 1:** Risk of bias assessment according to the Quality Assessment Tool for Qualitative Studies [53]. Ratings for each domain span from “1” to “3”, with “1” indicating “strong”, “2” “moderate”, and “3” “weak” quality. Summary assessment: studies without a “weak” rating in any of the domains received a global rating score of “strong”. Studies with only one “weak” rating received a global rating score of “moderate”. Studies with multiple “weak” ratings across domains received a global rating score of “weak”. Global ratings are highlighted in shades of grey with increased risk of bias corresponding to darker shades of grey.

Authors & Year	Selection Bias	Study Design	Confounds	Blinding	Collection Method	Withdrawals & Dropouts	Global Rating
Bruhl, et al. [55]	3	2	1	2	1	1	moderate
Canterberry, et al. [56]	3	2	1	2	2	1	moderate
Caria, et al. [57]	3	1	3	2	1	1	weak
Chung, et al. [58]	3	1	1	2	1	2	moderate
Gröne, et al. [59]	3	2	3	2	1	1	weak
Hamilton, et al. [60]	3	1	1	2	2	1	moderate
Hamilton et al. [61]	3	1	1	2	1	1	moderate
Hanlon, et al. [62]	3	2	1	2	2	2	moderate
Hartwell, et al. [63]	3	1	1	2	1	2	moderate
Hellrung, et al. [64]	3	1	2	2	1	1	moderate
Herwig, et al. [65]	3	1	1	2	1	2	moderate
Ihssen, et al. [66]	3	2	1	2	3	1	weak
Johnston, et al. [67]	3	1	3	2	1	1	weak
Johnston, et al. [68]	3	2	1	2	1	1	moderate
Karch, et al. [69]	2	1	3	2	1	1	moderate
Karch, et al. [70]	2	1	1	1	1	1	strong
Keller, et al. [71]	3	1	1	1	1	1	moderate
Kirschner, et al. [72]	2	1	2	3	1	1	moderate
Li, et al. [73]	3	2	1	2	1	1	moderate
Lieberman, et al. [74]	3	1	1	2	1	1	moderate
Linden, et al. [75]	3	1	1	3	1	1	weak
Liu, et al. [76]	3	1	3	2	1	1	weak
Marxen, et al. [77]	3	2	1	2	1	1	moderate
Mayeli, et al. [78]	3	1	3	2	2	1	weak
Mehler, et al. [79]	2	1	1	2	1	2	strong
Misaki, et al. [80]	3	1	2	1	1	1	moderate
Nicholson, et al. [25]	3	2	1	2	1	1	moderate
Nicholson, et al. [81]	3	2	1	2	1	1	moderate
Nicholson, et al. [82]	2	1	2	2	1	1	strong
Paret, et al. [83]	3	1	1	1	1	1	moderate
Paret, et al. [84]	3	2	1	2	1	1	moderate
Rana, et al. [85]	3	2	1	2	3	1	weak
Sarkheil, et al. [86]	3	1	3	2	1	1	weak
Scheinost, et al. [87]	3	1	1	2	2	1	moderate
Wang, et al. [88]	3	1	3	2	1	1	weak
Young, et al. [40]	3	1	1	1	1	1	moderate
Young, et al. [89]	3	1	1	1	1	1	moderate
Zhu, et al. [90]	3	1	1	2	1	1	moderate
Zotev, et al. [91]	3	1	1	3	1	3	weak
Zweerings, et al. [92]	2	1	1	2	1	1	strong
Zweerings, et al. [93]	2	1	1	2	1	1	strong

**Table 2:** Results from region of interest (ROI) analyses of included studies. Studies with both clinical and healthy samples are listed in the section of their respective clinical sample.

Authors & Year	Population (N)	Type of Control Group	Training ROI(s)	Modulation Direction in Experimental Condition	Results from ROI Analyses					
					Within Group Effect		Between Group Effect	Transfer Effect	Behavioural Effect	Brain–Behavioural Association
					Condition Effect	Training Effect				
*Studies with only healthy samples (N = 18)*										
Bruhl, Scherpiet, Sulzer, Stampfli, Seifritz and Herwig [55]	healthy (N = 6)	/	right amygdala	down	?	✓	/	/	/	/
Caria, Sitaram, Veit, Begliomini and Birbaumer [57]	healthy (N = 27)	CG 1: Sham (average) NFB CG 2: Emotional Imagery without NFB	left anterior insula	up (+ down in baseline blocks)	?	✓	x	/	✓ (aversive picture valence) x (negative picture arousal, positive picture arousal, valence)	✓ (aversive picture valence) x (negative picture arousal, positive picture arousal, valence)
Gröne, Dyck, Koush, Bergert, Mathiak, Alawi, Elliott and Mathiak [59]	healthy (N = 24)	/	rostral ACC	up	✓	✓	/	/	✓ (PANAS—subscales: mood) x (PANAS—global: mood, emotional prosody)	✓ (PANAS—subscales: mood) x (PANAS—global: mood, emotional prosody)
[61]	healthy (N = 17)	Sham (yoked) NFB	left or right subgenual ACC	down	✓	✓	✓	x (time, group)	/	/
Hellrung, Dietrich, Hollmann, Pleger, Kalberlah, Roggenhofer, Villringer and Horstmann [64]	healthy (N = 42)	No NFB	left amygdala	up and down	?	✓	✓	✓ (time, group)	/	/
Herwig, Lutz, Scherpiet, Scheerer, Kohlberg, Opialla, Preuss, Steiger, Sulzer, Weidt, Stampfli, Rufer, Seifritz, Jancke and Bruhl [65]	healthy (N = 26)	Sham (random) NFB	right amygdala	down	✓	✓	✓	x (time, group)	/	/
Johnston, Linden, Healy, Goebel, Habes and Boehm [67]	healthy (N = 31)	No NFB	left or right vlPFC/dlPFC, left, right or bilateral insula, right or bilateral medial temporal lobe, right inferior frontal gyrus	up	?	✓	✓	/	x (PANAS, POMS: mood)	?
Johnston, Boehm, Healy, Goebel and Linden [68]	healthy (N = 13)	/	unilateral or bilateral vlPFC/insula or medial temporal lobe/amygdala	up	✓	✓	/	/	? (described descriptively only)	?
Liu, Yao and Zhao [76]	healthy (N = 30)	No NFB	Left amygdala	up	?	✓	✓	/	/	/
Marxen, Jacob, Muller, Posse, Ackley, Hellrung, Riedel, Bender, Epple and Smolka [77]	healthy (N = 32)	/	bilateral amygdala	up and down	x	?	/	✓ (condition: up vs. down)	?	✓ (up- vs. downregulaton difference in arousal ratings) x (TAS—difficulties identifying feelings; susceptibility to anger)
Mayeli, Misaki, Zotev, Tsuchiyagaito, Al Zoubi, Phillips, Smith, Stewart, Refai, Paulus and Bodurka [78]	healthy (N = 27)	Sham (random) NFB	vmPFC	up	✓	?	x	?	/	/
Paret, Kluetsch, Ruf, Demirakca, Hoesterey, Ende and Schmahl [83]	healthy (N = 32)	Sham (control region) NFB	bilateral amygdala (EG); rostral basal ganglia area (CG)	down	x	x	x	✓ (condition)	? (picture arousal and valence ratings)	?
Paret, Zahringer, Ruf, Gerchen, Mall, Hendler, Schmahl and Ende [84]	healthy (N = 20)	/	amygdala	up and down	✓ (up vs. down)	?	/	/	? (picture arousal and valence ratings)	?
Sarkheil, Zilverstand, Kilian-Hutten, Schneider, Goebel and Mathiak [86]	healthy (N = 14)	No NFB	left lateral PFC	up	?	?	x	/	? (PANAS_ mood; picture arousal and valence ratings)	?
Scheinost, Stoica, Saksa, Papademetris, Constable, Pittenger and Hampson [87]	healthy (N = 20)	Sham (yoked) NFB	orbitofrontal cortex	up and down	?	✓	x	?	✓ (contamination anxiety)	✓
Wang, Yao and Zhao [88]	healthy (N = 30)	No NFB	left amygdala	up	?	✓	✓	/	x (PANAS: mood)	?
Zhu, Gao, Tong, Li, Wang, Zhang, Yang and Yan [90]	healthy (N = 26)	Sham (other region) NFB	hippocampus (EG), intraparietal sulcus (CG)	up	✓	✓	x	/	x (ERQ: emotion regulation, SAS: anxiety; SDS: depressive symptoms; PANAS: mood; HAMD: depressive symptoms)	?
Zotev, Krueger, Phillips, Alvarez, Simmons, Bellgowan, Drevets and Bodurka [91]	healthy (N = 28)	Sham (other region) NFB	left amygdala (EG); left horizontal segment of intraparietal sulcus (CG)	up	?	✓	✓	✓ (time, group)	? (TAS: alexithymia; ECS: emotional contagion)	✓ (difficulty identifying feelings subscale) x (difficulty describing, feelings, externally oriented thinking subscale, susceptibility to anger subscale)
*Studies with MDD samples (N = 6)*										
Hamilton, Glover, Bagarinao, Chang, Mackey, Sacchet and Gotlib [60]	MDD (N = 22)	Sham (yoked) NFB	fronto-insular cortex, dorsal ACC	down	?	✓	x	?	? (picture valence, self-referent encoding)	x (picture valence ratings, self-referent encoding)
Keller, Zweerings, Klasen, Zvyagintsev, Iglesias, Mendoza Quiñones and Mathiak [71]	MDD (N = 39)	HC	left and right vlPFC	up	?	?	x	/	✓ (BDI: depression) ? (emotion regulation, negative picture valence and arousal)	x (BDI: depressive symptoms)
	healthy (N = 37)				?	?		/		
Linden, Habes, Johnston, Linden, Tatineni, Subramanian, Sorger, Healy and Goebel [75]	MDD (N = 16)	No NFB	right/left vlPFC, left/right insula, left/right dlPFC, left/right prefrontal lobe, orbitofrontal cortex	up	✓	✓	?	/	✓ (HDRS: depressive symptoms) x (PANAS/POMS: mood)	✓ (HDRS: depressive symptoms)
Mehler, Sokunbi, Habes, Barawi, Subramanian, Range, Evans, Hood, Luhrs, Keedwell and et al. [79]	MDD (N = 32)	Sham (control region) NFB	fronto-limbic ROI (e.g., insula, striatum; EG); parahippocampal place area (CG)	up	✓	x	x	x (condition, time)	✓ (HDRS: depressive symptoms)	?
Young, Siegle, Zotev, Phillips, Misaki, Yuan, Drevets and Bodurka [40]	MDD (N = 33)	Sham (other region) NFB	amygdala (EG), left horizontal segment of intraparietal sulcus (CG)	up	✓	?	✓	?	✓ (MADRS/BDI-II/HDRS: depressive symptoms, SHAPS: pleasure; HAMA: anxiety)	✓ (MADRS: depressive symptoms) ? (HDRS: depressive symptoms; SHAPS: pleasure; HAMA: anxiety)
Young, Zotev, Phillips, Misaki, Yuan, Drevets and Bodurka [89]	MDD (N = 21)	Sham (other region) NFB	amygdala (EG), left horizontal segment of intraparietal sulcus (CG)	up	left amygdala: ✓ right amygdala: x	✓ x	✓ ✓	✓ (condition, group, time) ✓ (group)	✓ (POMS: depression, anger; STAI: anxiety; happiness, restlessness, anxiety, irritability) x (POMS: total, tension, fatigue, friendly, confused, vigour; sadness, drowsiness, alertness) ? (emotional contagion, TAS: alexithymia)	✓ (TAS—difficulties identifying feelings: alexithymia) ? (POMS: mood; STAI: anxiety; emotional contagion)
*Studies with PTSD samples (N = 6)*										
Lieberman, Rabellino, Densmore, Frewen, Steyrl, Scharnowski, Theberge, Neufeld, Schmahl, Jetly, Narikuzhy, Lanius and Nicholson [74]	PTSD (N = 14)	HC	PCC	down	?	?	x	?	✓ (PTSD reliving and distressing symptoms)	?
	healthy (N = 15)				?	?		?		
[80]	PTSD (N = 29)	Sham (control region) NFB	left amygdala (EG); left intraparietal sulcus (CG)	up	✓	x	?	?	Reported in different study	x (symptom change)
Nicholson, Rabellino, Densmore, Frewen, Paret, Kluetsch, Schmahl, Theberge, Ros, Neufeld, McKinnon, Reiss, Jetly and Lanius [81]	PTSD (N = 14)	/	bilateral amygdala	down	✓	✓	/	✓ (condition)	x (RSDI: PTSD symptoms)	?
Nicholson, Rabellino, Densmore, Frewen, Steryl, Scharnowski, Theberge, Neufeld, Schmahl, Jetly and Lanius [82]	PTSD (N = 14)	HC	PCC	down	✓	x	x	?	✓ (RSDI: reliving and distressing PTSD symptoms) ? (DERS: emotion regulation)	?
	healthy (N = 15)				x	x		?		
Zweerings, Pflieger, Mathiak, Zvyagintsev, Kacela, Flatten and Mathiak [92]	PTSD (N = 9)	HC	ACC	up	?	?	x	?	✓ (PANAS—positive affect: mood) x (PANAS—negative affect: mood)	?
	healthy (N = 9)				?	?		?		
Zweerings, Sarkheil, Keller, Dyck, Klasen, Becker, Gaebler, Ibrahim, Turetsky, Zvyagintsev and et al. [93]	PTSD (N = 20)	HC	left lateral PFC	up	x	?	x	/	✓ (PANAS: mood, ETI: PTSD symptoms) x (picture valence and arousal ratings)	✓ (PANAS: mood; ETI: PTSD symptoms) ? (picture arousal and valence ratings)
	healthy (N = 21)				x	?		/		
*Studies with substance use samples (N = 9)*										
Canterberry, Hanlon, Hartwell, Li, Owens, LeMatty, Prisciandaro, Borckardt, Saladin and Brady [56]	nicotine dependent smokers (N = 9)	/	ACC	up (localiser) + down	✓	x	/	/	✓ (craving ratings)	?
Chung, White, Geier, Johnston, Smyth, Delgado, McKee and Wilson [58]	nicotine-dependent smokers (N = 24)	No NFB	striatum/ caudate nucleus	up	✓	x	x	x (time, group)	x (QSU-B: smoking urge, SNW: smoking withdrawal, PANAS: affect valence, SSCCS: fatigue)	?
Hanlon, Hartwell, Canterberry, Li, Owens, LeMatty, Prisciandaro, Borckardt, Brady and George [62]	nicotine-dependent smokers (N = 15)	/	ventral ACC (craving reduction)	down	✓	?	/	/	✓ (craving)	✓
			dmPFC (craving resistance)	up	x	?	/	/	?	?
Hartwell, Hanlon, Li, Borckardt, Canterberry, Prisciandaro, Moran-Santa Maria, LeMatty, George and Brady [63]	nicotine-dependent smokers (N = 44)	No NFB	ACC (including, medial PFC and orbitofrontal cortex)	down	?	x	✓	/	?	?
Karch, Keeser, Hümmer, Paolini, Kirsch, Karali, Kupka, Rauchmann, Chrobok and Blautzik [69]	alcohol use disorder (N = 15)	Control group 1: Sham (yoked) NFB Control group 2: HC	ACC/dlPFC/insula	down	?	✓	GC1: x GC2: ?	/	x (OCDS: craving)	?
	healthy (N = 19)		PFC	down	?	x		/		
Karch, Krause, Lehnert, Konrad, Haller, Rauchmann, Maywald, Engelbregt, Adorjan and Koller [70]	alcohol use disorder (N = 48)	Sham (control region) NFB	ACC/dlPFC/insula (EG); Occipital region (CG)	down	?	x	x	/	✓ (BIS: impulsiveness; BDI: depressive symptoms; OCDS: craving) x (STAXI: anger; STAI: anxiety)	?
Kirschner, Sladky, Haugg, Stampfli, Jehli, Hodel, Engeli, Hosli, Baumgartner, Sulzer, Huys, Seifritz, Quednow, Scharnowski and Herdener [72]	cocaine users (N = 22)	HC	ventral tegmental area/substantia nigra	up	✓	x	x	?	/	/
	healthy (N = 28)				✓	x		?		
Li, Hartwell, Borckardt, Prisciandaro, Saladin, Morgan, Johnson, LeMatty, Brady and George [73]	nicotine-dependent smokers (N = 12)	/	ACC (craving reduction)	Down	✓	✓	/	/	✓ (craving)	?
			right middle PFC (craving resistance)	up	x	x	/	/	x (craving)	x
Rana, Ruiz, Corzo, Muehleck, Eck, Salinas, Zamorano, Silva, Rea and Batra [85]	nicotine-dependent smokers (N = 4)	Sham (random) NFB	bilateral anterior insula	down	?	?	?	✓ (time)	✓ (craving, cigarettes per day, QSU—B: smoking urge)	?

For some studies multiple results were considered for syntheses due to additional samples or paradigms. The second set of considered results within one study is marked in grey. Significant: “✓”; not significant: “x”. Calculation of effect was not possible due to lack of data: “/”. No reporting of a specific effect despite availability of respective data: “?”. Condition effect: rt-fMRI-NFB regulate-condition versus non-regulate condition; training effect: pre-post rt-fMRI-NFB difference, or first versus last run difference, or baseline versus last run difference; group effect: experimental/clinical versus control/healthy group; transfer effect: rt-fMRI-NFB specific effect during transfer run; behavioural effect: pre-post rt-fMRI-NFB improvement in clinical outcome measure; brain–behavioural association: correlation between rt-fMRI-NFB specific brain response and behavioural measures. ALS = Affect Liability Scale; ACC = anterior cingulate cortex; BDI = Beck Depression Inventory; BIS = Barrat Impulsiveness Scale; CG = control group (no rt-fMRI-NF intervention); DERS = Difficulties in Emotion Regulation Scale; dlPFC = dorsolateral prefrontal cortex; dmPFC = dorsomedial prefrontal cortex; DTS—short = Dissociation-Tension Scale; ECS = Emotional Contagion Scale; EG = experimental/clinical group (with rt-fMRI-NF intervention); ERG = Emotion Regulation Questionnaire; FSQ = Fear of Spiders Questionnaire; ETI = Essener Trauma-Inventar; HAMA = Hamilton Anxiety Rating Scale; HAMD = Hamilton Depression Scale; HC = healthy controls/group; HDRS = Hamilton Depression Rating Scale; MDD = major depressive disorder; OCDS = Obsessive-Compulsive Drinking Scale; PANAS = Positive and Negative Affect Scale; PCC = posterior cingulate cortex; POMS = Profile of Mood States; PTSD = post-traumatic stress disorder; QSU: Questionnaire of Smoking Urges; QSU-B: Questionnaire of Smoking Urges-Brief; RSDI = Response-Driven Imagery Scale; SAS = Self-Rating Anxiety Scale; SDS = Self-report Depression Scale; SEK = Emotion Regulation Skill Questionnaire; Sham (average) NFB = active control group in which the NFB signal was based on the average whole brain activation of the participant; Sham (other region) NFB = active control group in which the NFB signal was based on a brain region other than the ROI; Sham (random) NFB = active control group in which the NFB signal was based on a random pattern; Sham (yoked) NFB = active control group in which the NFB signal was based on the ROI brain activation of a different participant; SHAPS = Snaith-Hamilton Pleasure Scale; SSCCS: State Self-Control Capacity Scale; SNW: Symptoms of Nicotine Withdrawal Scale; STAXI = State-Trait Anger Expression Inventory; STAI = State-Trait Anxiety Inventory; TAS = Toronto Alexithymia Scale; vlPFC = ventrolateral prefrontal cortex; vmPFC = ventromedial prefrontal cortex; ZAN-BPD = Zanari Rating Scale for BPD.

**Table 3:** The frequency of significant ROI effects for all samples, healthy samples, and clinical samples for *condition effect*, *training effect*, *group effect*, *transfer effect*, *behavioural effect*, and *brain–behavioural association*. For each contrast, the number of results theoretically available based on the study design (N_theoretical_) and the number of significant results (N_signf_) are presented in brackets. As some studies included both healthy and clinical samples, results for both samples were considered for the calculation of *condition effect*, *training effect* and *transfer effect*. N_studies_ defines the number of studies for each population from which data has been included for syntheses.

Percent of Significant Results	All Samples (N_studies_ = 39)	Healthy Samples (N_studies_ = 25)	Clinical Samples (N_studies_ = 21)
100%			
76–99%			**behavioural effect** (N_theoretical_ = 19, N_signf_ = 14)
51–75%	**behavioural effect** (N_theoretical_ = 31, N_signf_ = 17)	**training effect **(N_theoretical_ = 25, N_signf_ = 13)	**condition effect** (N_theoretical_ = 23, N_signf_ = 12)
26–50%	**condition effect** (N_theoretical_ = 48, N_signf_ = 20); **training effect** (N_theoretical_ = 48, N_signf_ = 19); **group effect** (N_theoretical_ = 31, N_signf_ = 10); **transfer effect** (N_theoretical_ = 24, N_signf_ = 7); **brain-behaviour-association** (N_theoretical_ = 32, N_signf_ = 10)	**condition effect** (N_theoretical_ = 25, N_signf_ = 8); **group effect** (N_theoretical_ = 14, N_signf_ = 7); **transfer effect** (N_theoretical_ = 12, N_signf_ = 4); **brain–behavioural-association** (N_theoretical_ = 12, N_signf_ = 5)	**training effect** (N_theoretical_ = 23, N_signf_ = 6)
1–25%		**behavioural effect** (N_theoretical_ = 12, N_signf_ = 3)	**group effect** (N_theoretical_ = 17, N_signf_ = 3); **transfer effect** (N_theoretical_ = 12, N_signf_ = 3); **brain-behaviour-association** (N_theoretical_ = 20, N_signf_ = 5)
0%			

**Table 4:** The frequency of significant region of interest (ROI) effects for clinical samples diagnosed with MDD, PTSD, and substance use, respectively, for the *condition effect*, *training effect*, *group effect*, *transfer effect*, *behavioural effect*, and *brain–behavioural association*. For each contrast, the number of results theoretically available based on the study design (N_theoretical_) and the number of significant results (N_signf_) are presented in brackets, followed by the references of the studies that reported those significant results. Results for the *brain–behavioural association* were not reported for PTSD and substance use samples, and *transfer effect* was not reported for any of the three groups as less than three results were available for each respective contrast. N_studies_ defines the number of studies for each population from which data has been included for syntheses. Abbreviations: MDD = Major Depressive Disorder; PTSD = Post-traumatic Stress Disorder.

Percent of Significant Results	MDD (N_studies_ = 6)	PTSD (N_studies_ = 6)	Substance Use (N_studies_ = 9)
100%			
76–99%	**behavioural effect** (N_theoretical_ = 6, N_signf_ = 5)	**behavioural effect **(N_theoretical_ = 5, N_signf_ = 4)	
51–75%	**condition effect** (N_theoretical_ = 6, N_signf_ = 4)		**behavioural effect** (N_theoretical_ = 8, N_signf_ = 5)
26–50%	**training effect** (N_theoretical_ = 6, N_signf_ = 3) **group effect** (N_theoretical_ = 6, N_signf_ = 2) **brain–behavioural association** (N_theoretical_ = 6, N_signf_ = 3)	**condition effect** (N_theoretical_ = 6, N_signf_ = 3)	**condition effect** (N_theoretical_ = 11, N_signf_ = 5) **transfer effect** (N_theoretical_ = 3, N_signf_ = 1)
1–25%	**transfer effect** (N_theoretical_ = 4, N_signf_ = 1)	**training effect** (N_theoretical_ = 6, N_signf_ = 1) **transfer effect** (N_theoretical_ = 5, N_signf_ = 1) **brain–behavioural-association** (N_theoretical_ = 6, N_signf_ = 1)	**training effect** (N_theoretical_ = 11, N_signf_ = 2) **group effect** (N_theoretical_ = 6, N_signf_ = 1) **brain–behavioural association** (N_theoretical_ = 8, N_signf_ = 1)
0%		**group effect** (N_theoretical_ = 5; N_signf_ = 0)	

**Table 5:** The frequency of significant effects for the rt-fMRI-NFB training regions of interest (ROIs) “amygdala”, “PFC”, “individualised multi-region ROIs”, and “other ROIs”. For each contrast the number of results theoretically available based on the study design (N_theoretical_) and the number of significant results (N_signf_) are presented in brackets, followed by the references of the studies that reported those significant results. Studies with multiple rt-fMRI-NFB paradigms, or both clinical and healthy samples, were considered separately for *condition effects*, *training effects*, and *transfer effects*. Due to the lack of sufficient data, the *training effect*, *behavioural effect*, and *brain–behavioural association* are not reported for the PFC ROI. The *transfer effect* was not reported for PFC and protocols using individualised multi-region ROIs. The following brain regions were included in the category of “other ROI”: ACC (N = 5), anterior insula (N = 2), hippocampus (N = 1), orbitofrontal cortex (N = 1), PCC (N = 2), mesolimbic areas (N = 2). Individualised multi-region ROIs included either the amygdala, PFC, or those regions listed under the “other ROI” category. N_studies_ defines the number of studies for each population from which data has been included for syntheses. Abbreviations: NFB = neurofeedback; PFC = prefrontal cortex, ROI = region of Interest.

Percent of Significant Results	Amygdala (N_studies_ = 13)	PFC (N_studies_ = 4)	Individualised Multi-Region ROIs (N_studies_ = 9)	Other ROIs (N_studies_ = 13)
100%				
76–99%	**group effect** (N_theoretical_ =9; N_signf_ = 7)			
51–75%	**training effect** (N_theoretical_ = 13; N_signf_ = 8) **transfer effect** (N_theoretical_ = 9; N_signf_ = 6)		**behavioural effect** (N_theoretical_ = 9; N_signf_ = 5)	**behavioural effect** (N_theoretical_ = 11; N_signf_ = 8)
26–50%	**condition effect** (N_theoretical_ = 13; N_signf_ = 6) **brain–behavioural association** (N_theoretical_ = 9; N_signf_ = 4)		**condition effect** (N_theoretical_ = 12; N_signf_ = 5) **training effect** (N_theoretical_ = 12; N_signf_ = 6)	**condition effect** (N_theoretical_ = 17; N_signf_ = 8) **training effect** (N_theoretical_ = 17; N_signf_ = 5) **brain–behavioural association **(N_theoretical_ = 11; N_signf_ = 3)
1–25%	**behavioural effect** (N_theoretical_ = 8; N_signf_ = 2)	**condition effect** (N_theoretical_ = 6; N_signf_ = 1)	**group effect** (N_theoretical_ = 7; N_signf_ = 1) **brain–behavioural association** (N_theoretical_ = 9; N_signf_ = 2)	**group effect** (N_theoretical_ = 11; N_signf_ = 2) **transfer effect** (N_theoretical_ = 12; N_signf_ = 1)
0%		**training effect** (N_theoretical_ = 6; N_signf_ = 0) **group effect** (N_theoretical_ = 4; N_signf_ = 0)		

**Table 6:** Whole-brain results. Significant: “✓”; not significant: “x”. Calculation of effect was not possible due to lack of data: “/”. No reporting of a specific effect despite availability of respective data: “?”.

Authors & Year	Population (N)	Type of Control Group	Training ROI (s)	Modulation Direction in Experimental Condition	Results from Whole-Brain Analyses					
					Within Group Effect		Between Group Effect	Transfer Effect	Behavioural Effect	Brain–Behavioural Association
					Condition Effect	Training Effect				
*Studies with only healthy samples (N = 12)*										
Caria, Sitaram, Veit, Begliomini and Birbaumer [57]	Healthy (N = 27)	Sham (average) NFB	left anterior insula	up and down	?	✓	x	/	✓ (aversive picture valence) x (negative picture arousal, positive picture arousal, valence)	/
Gröne, Dyck, Koush, Bergert, Mathiak, Alawi, Elliott and Mathiak [59]	Healthy (N = 24)	/	rostral ACC	up	✓	?	/	/	✓ (PANAS—subscales: mood) x (PANAS—global: mood, emotional prosody)	/
Hellrung, Dietrich, Hollmann, Pleger, Kalberlah, Roggenhofer, Villringer and Horstmann [64]	Healthy (N = 42)	No NFB	left amygdala	up and down	✓	?	?	?	/	/
Herwig, Lutz, Scherpiet, Scheerer, Kohlberg, Opialla, Preuss, Steiger, Sulzer, Weidt, Stampfli, Rufer, Seifritz, Jancke and Bruhl [65]	Healthy (N = 26)	Sham (random) NFB	right amygdala	down	?	✓	✓	?	/	/
Ihssen, Sokunbi, Lawrence, Lawrence and Linden [66]	Healthy (N = 10)	/	amygdala/basal ganglia/thalamus	down	✓	?	/	/	✓ (hunger ratings) x (craving; fullness ratings; picture motivational intensity; picture valence; state craving; trait craving)	/
Johnston, Linden, Healy, Goebel, Habes and Boehm [67]	Healthy (N = 31)	No NFB	left or right vlPFC, left or right dlPFC, left, right or bilateral insula, right or bilateral medial temporal lobe, right inferior frontal gyrus	up	✓	✓	?	/	x (PANAS, POMS: mood)	/
Johnston, Boehm, Healy, Goebel and Linden [68]	Healthy (N = 13)	/	unilateral or bilateral vlPFC/insula or medial temporal lobe/amygdala	up	?	✓	/	/	? (described descriptively only)	/
Marxen, Jacob, Muller, Posse, Ackley, Hellrung, Riedel, Bender, Epple and Smolka [77]	Healthy (N = 32)	/	bilateral amygdala	up and down	x (up vs. down)	?	/	?	?	/
Mayeli, Misaki, Zotev, Tsuchiyagaito, Al Zoubi, Phillips, Smith, Stewart, Refai, Paulus and Bodurka [78]	Healthy (N = 27)	Sham (random) NFB	vmPFC	up	✓	✓	?	?	/	/
Paret, Kluetsch, Ruf, Demirakca, Hoesterey, Ende and Schmahl [83]	Healthy (N = 32)	Sham (other region) NFB	bilateral amygdala (EG); rostral basal ganglia area (CG)	down	✓	?	x	x (group) ✓ (condition)	? (picture arousal and valence ratings)	/
Wang, Yao and Zhao [88]	Healthy (N = 30)	No NFB	left amygdala	up	?	✓	✓	/	x (PANAS: mood)	/
Zotev, Krueger, Phillips, Alvarez, Simmons, Bellgowan, Drevets and Bodurka [91]	Healthy (N = 28)	Sham (other region) NFB	left amygdala (EG); left horizontal segment of intraparietal sulcus (CG);	up	?	?	x	✓ (condition)	? (TAS: alexithymia; ECS: emotional contagion)	/
*Studies with MDD samples (N = 4)*										
Keller, Zweerings, Klasen, Zvyagintsev, Iglesias, Mendoza Quiñones and Mathiak [71]	MDD (N = 39)	HC	left and right vlPFC	up	x	✓	✓	/	✓ (BDI: depression) ? (emotion regulation, negative picture valence and arousal)	/
	Healthy (N = 37)				x	✓		/		
Linden, Habes, Johnston, Linden, Tatineni, Subramanian, Sorger, Healy and Goebel [75]	MDD (N = 16)	No NFB	right/left vlPFC, left/right insula, left/right dlPFC, left/right prefrontal lobe, orbitofrontal cortex	up	✓	✓	?	/	✓ (HDRS: depressive symptoms) x (PANAS/POMS: mood)	/
Mehler, Sokunbi, Habes, Barawi, Subramanian, Range, Evans, Hood, Luhrs, Keedwell and et al. [79]	MDD (N = 32)	Sham (other region) NFB	fronto-limbic ROI (e.g., insula, striatum; EG); parahippocampal place area (CG)	up	✓	?	✓	?	✓ (HDRS: depressive symptoms)	/
Young, Zotev, Phillips, Misaki, Yuan, Drevets and Bodurka [89]	MDD (N = 21)	Sham (other region) NFB	amygdala (EG), left horizontal segment of intraparietal sulcus (CG)	up	✓	?	✓	?	✓ (POMS: depression, anger; STAI: anxiety; happiness, restlessness, anxiety, irritability) x (POMS: total, tension, fatigue, friendly, confused, vigour; sadness, drowsiness, alertness) ? (emotional contagion, TAS: alexithymia)	/
*Studies with PTSD samples (N = 4)*										
[25]	PTSD (N = 10)	/	Amygdala	down	?	✓	/	x (time)	x (RSDI: PTSD symptoms)	✓ (state dissociation)
Nicholson, Rabellino, Densmore, Frewen, Steryl, Scharnowski, Theberge, Neufeld, Schmahl, Jetly and Lanius [82]	PTSD (N = 14)	HC	PCC	down	✓	?	✓	x (group)	✓ (RSDI: reliving and distressing PTSD symptoms) ? (DERS: emotion regulation)	/
	healthy (N = 15)				✓	?		?		
Zweerings, Pflieger, Mathiak, Zvyagintsev, Kacela, Flatten and Mathiak [92]	PTSD (N = 9)	HC	ACC	up	✓	✓	✓	✓ (condition) x (group)	✓ (PANAS—positive affect: mood) x (PANAS—negative affect: mood)	/
	healthy (N = 9)				✓	✓		✓ (condition)		
Zweerings, Sarkheil, Keller, Dyck, Klasen, Becker, Gaebler, Ibrahim, Turetsky, Zvyagintsev and et al. [93]	PTSD (N = 20)	HC	left lateral PFC	up	✓	?	x	/	✓ (PANAS: mood, ETI: PTSD symptoms) x (picture valence and arousal ratings)	/
	healthy (N = 21)				✓	?		/		
*Studies with substance use samples (N = 5)*										
Chung, White, Geier, Johnston, Smyth, Delgado, McKee and Wilson [58]	nicotine-dependent smokers (N = 44)	No NFB	striatum/ caudate nucleus	up	?	✓	✓	?	x (QSU-B: smoking urge, SNW: smoking withdrawal, PANAS: affect valence, SSCCS: fatigue)	/
Karch, Keeser, Hümmer, Paolini, Kirsch, Karali, Kupka, Rauchmann, Chrobok and Blautzik [69]	alcohol use disorder (N = 15)	CG 1: Sham (yoked) NFB CG 2: HC	ACC/dlPFC/insula	down	?	✓	?	/	x (OCDS: craving)	/
	healthy (N = 19)		PFC	down	?	✓		/		
Karch, Krause, Lehnert, Konrad, Haller, Rauchmann, Maywald, Engelbregt, Adorjan and Koller [70]	alcohol use disorder (N = 48)	Sham (other region) NFB	ACC/dlPFC/insula	down	?	✓	?	/	✓ (BIS: impulsiveness; BDI: depressive symptoms; OCDS: craving) x (STAXI: anger; STAI: anxiety)	/
Kirschner, Sladky, Haugg, Stampfli, Jehli, Hodel, Engeli, Hosli, Baumgartner, Sulzer, Huys, Seifritz, Quednow, Scharnowski and Herdener [72]	cocaine users (N = 22)	HC	ventral tegmental area/substantia nigra	up	✓	?	x	?	/	/
	healthy (N = 28)				✓	?		?		
Li, Hartwell, Borckardt, Prisciandaro, Saladin, Morgan, Johnson, LeMatty, Brady and George [73]	nicotine-dependent smokers (N = 12)	/	ACC (craving reduction)	Down	x	?	/	/	✓ (craving)	/
			right middle PFC (craving resistance)	up	x	?		/	x (craving)	/

**Cells highlighted in grey indicate multiple subgroups within one study.** Condition effect: rt-fMRI-NFB regulate-condition versus non-regulate condition; training effect: pre-post rt-fMRI-NFB difference, or first versus last run difference, or baseline versus last run difference; group effect: experimental/clinical versus control/healthy group; transfer effect: rt-fMRI-NFB specific effect during transfer run; behavioural effect: pre-post rt-fMRI-NFB improvement in clinical outcome measure; brain–behavioural association: correlation between rt-fMRI-NFB specific brain response and behavioural measures. CG = control group (no rt-fMRI-NFB intervention); EG = experimental/clinical group (with rt-fMRI-NFB intervention); HC = healthy controls/group; MDD = major depressive disorder; PTSD = post-traumatic stress disorder; Sham (average) NFB = active control group in which the NFB signal was based on the average whole brain activation of the participant; Sham (other region) NFB = active control group in which the NFB signal was based on a brain region other than the ROI; Sham (random) NFB = active control group in which the NFB signal was based on a random pattern; Sham (yoked) NFB = active control group in which the NFB signal was based on the ROI brain activation of a different participant.

**Table 7:** The frequency of significant whole-brain effects for all samples, healthy samples, and clinical samples for the *condition effect*, *training effect*, *group effect*, *transfer effect*, *behavioural effect*, and *brain–behavioural association*. For each contrast, the number of results theoretically available based on the study design (N_theoretical_) and the number of significant results (N_signf_) are presented in brackets, followed by the references of the studies that reported those significant results. As some studies included both healthy and clinical samples, results for both samples have been considered for calculation of *condition effect*, *training effect* and *transfer effect*. Results for *transfer effect* and *brain–behavioural association* could not be calculated due to the lack of at least three available results for these contrasts. N_studies_ defines the number of studies for each population from which data has been included for syntheses.

Percent of Significant Results	All Samples (N_studies_ = 25)	Healthy Samples (N_studies_ = 18)	Clinical Samples (N_studies_ = 13)
100%			
76–99%			
51–75%	**condition effect** (N_theoretical_ = 32; N_signf_ = 17) **behavioural effect** (N_theoretical_ = 21; N_signf_ = 12)	**condition effect** (N_theoretical_ = 18; N_signf_ = 10)	**group effect** (N_theoretical_ = 11; N_signf_ = 6) **behavioural effect** (N_theoretical_ = 12; N_signf_ = 9)
26–50%	**training effect** (N_theoretical_ = 32; N_signf_ = 16) **group effect** (N_theoretical_ = 19; N_signf_ = 8)	**training effect** (N_theoretical_ = 18; N_signf_ = 9) **transfer effect** (N_theoretical_ = 9; N_signf_ = 3) **behavioural effect** (N_theoretical_ = 9; N_signf_ = 3)	**condition effect** (N_theoretical_ = 14; N_signf_ = 7) **training effect** (N_theoretical_ = 14; N_signf_ = 7)
1–25%	**transfer effect** (N_theoretical_ = 16; N_signf_ = 4)	**group effect** (N_theoretical_ = 8; N_signf_ = 2)	**transfer effect** (N_theoretical_ = 7; N_signf_ = 1)
0%			

## Data Availability

To ensure transparency and reproducibility, we will make the search strategy, the literature hits, the study quality assessment, and the data synthesis protocol accessible to the public on OSF (https://osf.io/9ud5q/?view_only=c3519d1f13f143a4b316255063e7468c, accessed on 10 July 2024).

## Supplementary Materials

The following supporting information can be downloaded at: https://www.mdpi.com/article/10.3390/brainsci14070700/s1, Table S1: PRISMA Checklist; Table S2: Demographic data of included study samples; Table S3: The frequency of significant ROI effects for all samples, healthy samples, and clinical samples; Table S4: The frequency of significant region of interest (ROI) effects for clinical samples diagnosed with MDD, PTSD, and substance use; Table S5: The frequency of significant effects for the rt-fMRI-NFB training regions of interest (ROIs) “amygdala”, “PFC”, “individualized multi-region ROIs”, and “other ROIs”; Table S6: The frequency of significant whole-brain effects for all samples, healthy samples, and clinical samples.
