# Supplementary material for: The Clinical Impact of Real-Time fMRI Neurofeedback on Emotion Regulation: A Systematic Review
Source: Brain Sci. 2024 Jul 12;14(7):700. doi: 10.3390/brainsci14070700 (PMC11274904; doi:10.3390/brainsci14070700)
Supplement: Supplementary file 1 [file brainsci-14-00700-s001.zip › brainsci-3082518-supplementary.pdf]

Supplementary

S1: PRISMA Checklist

Section and Topic	Item #	Checklist item	Location where item is reported
**TITLE**			
Title	1	Identify the report as a systematic review.	1
**ABSTRACT**			
Abstract	2	See the PRISMA 2020 for Abstracts checklist.	2
**INTRODUCTION**			
Rationale	3	Describe the rationale for the review in the context of existing knowledge.	3–4
Objectives	4	Provide an explicit statement of the objective(s) or question(s) the review addresses.	4
**METHODS**			
Eligibility criteria	5	Specify the inclusion and exclusion criteria for the review and how studies were grouped for the syntheses.	5
Information sources	6	Specify all databases, registers, websites, organisations, reference lists and other sources searched or consulted to identify studies. Specify the date when each source was last searched or consulted.	5–6
Search strategy	7	Present the full search strategies for all databases, registers and websites, including any filters and limits used.	5–6
Selection process	8	Specify the methods used to decide whether a study met the inclusion criteria of the review, including how many reviewers screened each record and each report retrieved, whether they worked independently, and if applicable, details of automation tools used in the process.	6–7
Data collection process	9	Specify the methods used to collect data from reports, including how many reviewers collected data from each report, whether they worked independently, any processes for obtaining or confirming data from study investigators, and if applicable, details of automation tools used in the process.	6
Data items	10a	List and define all outcomes for which data were sought. Specify whether all results that were compatible with each outcome domain in each study were sought (e.g. for all measures, time points, analyses), and if not, the methods used to decide which results to collect.	6
	10b	List and define all other variables for which data were sought (e.g. participant and intervention characteristics, funding sources). Describe any assumptions made about any missing or unclear information.	/
Study risk of bias assessment	11	Specify the methods used to assess risk of bias in the included studies, including details of the tool(s) used, how many reviewers assessed each study and whether they worked independently, and if applicable, details of automation tools used in the process.	7–8
Effect measures	12	Specify for each outcome the effect measure(s) (e.g. risk ratio, mean difference) used in the synthesis or presentation of results.	8
Synthesis methods	13a	Describe the processes used to decide which studies were eligible for each synthesis (e.g. tabulating the study intervention characteristics and comparing against the planned groups for each synthesis (item #5)).	8
	13b	Describe any methods required to prepare the data for presentation or synthesis, such as handling of missing summary statistics, or data conversions.	8
	13c	Describe any methods used to tabulate or visually display results of individual studies and syntheses.	8
	13d	Describe any methods used to synthesize results and provide a rationale for the choice(s). If meta-analysis was performed, describe the model(s), method(s) to identify the presence and extent of statistical heterogeneity, and software package(s) used.	8
	13e	Describe any methods used to explore possible causes of heterogeneity among study results (e.g. subgroup analysis, meta-regression).	8
	13f	Describe any sensitivity analyses conducted to assess robustness of the synthesized results.	/
Reporting bias assessment	14	Describe any methods used to assess risk of bias due to missing results in a synthesis (arising from reporting biases).	/
Certainty assessment	15	Describe any methods used to assess certainty (or confidence) in the body of evidence for an outcome.	/
**RESULTS**			

Section and Topic	Item #	Checklist item	Location where item is reported
Study selection	16a	Describe the results of the search and selection process, from the number of records identified in the search to the number of studies included in the review, ideally using a flow diagram.	8-9
	16b	Cite studies that might appear to meet the inclusion criteria, but which were excluded, and explain why they were excluded.	/
Study characteristics	17	Cite each included study and present its characteristics.	11–16, supplementary
Risk of bias in studies	18	Present assessments of risk of bias for each included study.	9–11
Results of individual studies	19	For all outcomes, present, for each study: (a) summary statistics for each group (where appropriate) and (b) an effect estimate and its precision (e.g. confidence/credible interval), ideally using structured tables or plots.	11–16; 21–23
Results of syntheses	20a	For each synthesis, briefly summarise the characteristics and risk of bias among contributing studies.	/
	20b	Present results of all statistical syntheses conducted. If meta-analysis was done, present for each the summary estimate and its precision (e.g. confidence/credible interval) and measures of statistical heterogeneity. If comparing groups, describe the direction of the effect.	16–21; 23–24
	20c	Present results of all investigations of possible causes of heterogeneity among study results.	19–21
	20d	Present results of all sensitivity analyses conducted to assess the robustness of the synthesized results.	/
Reporting biases	21	Present assessments of risk of bias due to missing results (arising from reporting biases) for each synthesis assessed.	/
Certainty of evidence	22	Present assessments of certainty (or confidence) in the body of evidence for each outcome assessed.	/
**DISCUSSION**			
Discussion	23a	Provide a general interpretation of the results in the context of other evidence.	24–26
	23b	Discuss any limitations of the evidence included in the review.	26,
	23c	Discuss any limitations of the review processes used.	26
	23d	Discuss implications of the results for practice, policy, and future research.	27
**OTHER INFORMATION**			
Registration and protocol	24a	Provide registration information for the review, including register name and registration number, or state that the review was not registered.	5
	24b	Indicate where the review protocol can be accessed, or state that a protocol was not prepared.	/
	24c	Describe and explain any amendments to information provided at registration or in the protocol.	5
Support	25	Describe sources of financial or non-financial support for the review, and the role of the funders or sponsors in the review.	/
Competing interests	26	Declare any competing interests of review authors.	1
Availability of data, code and other materials	27	Report which of the following are publicly available and where they can be found: template data collection forms; data extracted from included studies; data used for all analyses; analytic code; any other materials used in the review.	5

S2: demographic data of included study samples

Authors & year	Population	Psychiatric diagnoses	Sample size	Mean age/age range in years (SD)	Percentage of female participants
Studies with only healthy samples (N = 19)					
Bruhl et al. [1]	healthy	/	6	26 (3.8)	67
Caria et al. [2]	healthy	/	27	27.51	56
Gröne et al. [3]	healthy	/	24	26.48*	33
Hamilton et al. [4]	healthy	/	17	29,84*	100
Hellrung et al. [5]	healthy	/	42	26.87*	0
Herwig et al. [6]	healthy	/	26	26.95*	50
Ihssen et al. [7]	healthy	/	10	21.4 (2.3)	100
Johnston et al. [8]	healthy	/	31	21–54	52
Johnston et al. [9]	healthy	/	13	21–52	69
Liu et al. [10]	healthy	/	30	23.37*	53
Marxen et al. [11]	healthy	/	32	24.7	47
Mayeli et al. [12]	healthy	/	27	29	48
Paret et al. [13]	healthy	/	32	24.57*	100
Paret et al. [14]	healthy	/	20	24.57 (4.45)	100

Sarkheil et al. [15]	healthy	/	14	20–27	57
Scheinost et al. [16]	healthy (high contamination anxiety)	/	20		40
Wang et al. [17]	healthy	/	30	23.37*	53
Zhu et al. [18]	healthy	/	26	23.2 (1.4)	54
Zotev et al. [19]	healthy	/	28	28.0 (9.0)	100
Studies with MDD samples (N = 6)					
Hamilton et al. [20]	clinical	MDD	22	32,85*	100
Keller et al. [21]	clinical	MDD	39	35.2 (2.2)	44
	healthy		37	32.3 (2.1)	41
Linden et al. [22]	clinical	MDD	16	48.44*	19
Mehler et al. [23]	clinical	MDD	32	47.07*	66
Young et al. [24]	clinical	MDD	33	31.53*	72
Young et al. [25]	clinical	MDD	21	37.78*	86
Studies with PTSD samples (N = 7)					
Lieberman et al. [26]	clinical	PTSD	14	49.5 (5.11)	43
	healthy		15	37.73 (12.86)	67
Misaki et al. [27]	clinical	PTSD	29	31.55*	0
Nicholson et al. [28]	clinical	PTSD	10	49.6 (6.5)	60

Nicholson et al. [29]	clinical	PTSD	14	48.1 (9.8)	64
Nicholson et al. [30]	clinical	PTSD	14	49.5 (5.11)	43
	healthy		15	37.73 (12.86)	67
Zweerings et al. [31]	clinical	PTSD	9	42.3 (14.1)	89
	healthy		9	41.3 (13.1)	
Zweerings et al. [32]	clinical	PTSD	20	45.5 (12.2)	40
	healthy		21	44.1 (10.9)	43
Studies with substance use samples (N = 9)					
Canterberry et al. [33]	clinical	nicotine dependent smokers	9	32.7 (13.01)	11
Chung et al. [34]	clinical	nicotine-dependent smokers	44	26.7 (7.5)	23
Hanlon et al. [35]	clinical	nicotine-dependent smokers	15	21–45	/
Hartwell et al. [36]	clinical	nicotine-dependent smokers	44	35.2*	36
Karch et al. [37]	clinical	alcohol use disorder	15	18–60	/
	healthy		19		
Karch et al. [38]	clinical	alcohol use disorder	48	45.5*	14
Kirschner et al. [39]	clinical	cocaine users	22	28.97 *	44
	healthy		28		
Li et al. [40]	clinical	nicotine-dependent smokers	12	28.7 (10.9)	67
Rana et al. [41]	clinical	nicotine-dependent smokers	4		

Supplementary table 2: [29] includes a subsample already published in [28]. Both mean ages and percentage of female participants occasionally were calculated manually by the authors of this review based on the available age- and gender-related information reported in the respective studies. Manually calculated mean ages are noted with “*”. Manually calculated percentages of female participants were rounded to integers.

S3: The frequency of significant ROI effects for all samples, healthy samples, and clinical samples

Percentage of significant results	All samples ($N_{studies}$ = 39)	Healthy samples ($N_{studies}$ = 25)	Clinical samples ($N_{studies}$ = 21)
100%			
76–99%			**behavioural effect** [$N_{theoretical}$ = 19, N_{signf} = 14; 21–26,30–32,33,35,38,40,41]
51–75%	**behavioural effect** [$N_{theoretical}$ = 31; N_{signf} = 17; 2,3,16,21–26,30–32,33,35,38,40,41]	**training effect** [$N_{theoretical}$ = 25; N_{signf} = 13; 1,2–6,8–10,16–19]	**condition effect** [$N_{theoretical}$ = 23; N_{signf} = 12; 22–25,27,29,30,33,34,35,39,40]
26–50%	**condition effect** [3,4,6,9,12,14,18,22–25,27,29,30, $N_{theoretical}$ = 48; N_{signf} = 20; 33,34,35,2x 39,40]<br>**training effect** [$N_{theoretical}$ = 48; N_{signf} = 19; 1,2–6,8–10,16–20,22,25,29,37,40]<br>**group effect** [$N_{theoretical}$ = 31; N_{signf} = 10; 4,5,6,8,10,17,19,24,25,36]<br>**transfer effect** [$N_{theoretical}$ = 24; N_{signf} = 7; 5,11,13,19,25,29,41]<br>**brain–behaviour association** [$N_{theoretical}$ = 32; N_{signf} = 10; 2,3,11,16,19,22,24,25,32,35]	**condition effect** [$N_{theoretical}$ = 25; N_{signf} = 8; 3,4,6,9,12,14,18,39]<br>**group effect** [$N_{theoretical}$ = 14; N_{signf} = 7; 4,5,6,8,10,17,19]<br>**transfer effect** [$N_{theoretical}$ = 12; N_{signf} = 4; 5,11,13,19]<br>**brain–behavioural association** [$N_{theoretical}$ = 12; N_{signf} = 5; 2,3,11,16,19]	**training effect** [$N_{theoretical}$ = 23; N_{signf} = 6; 20,22,25,29,37,40]
1–25%		**behavioural effect** [$N_{theoretical}$ = 12; N_{signf} = 3; 2,3,16]	**group effect** [$N_{theoretical}$ = 17; N_{signf} = 3; 24,25,36],<br>**transfer effect** [$N_{theoretical}$ = 12; N_{signf} = 3; 25,29,41],<br>**brain–behaviour association** [$N_{theoretical}$ = 20; N_{signf} = 5; 22,24,25,32,35]
0%			

Supplementary table 3. The frequency of significant ROI effects for all samples, healthy samples, and clinical samples for *condition effect*, *training effect*, *group effect*, *transfer effect*, *behavioural effect*, and *brain–behavioural association*. For each contrast the number of results theoretically available based on the study design ($N_{theoretical}$) and the number of significant results (N_{signf}) are presented in brackets, followed by the references of the studies that reported those significant results. As some studies included both healthy and clinical samples, results for both samples have been considered for calculation of *condition effect*, *training effect* and *transfer effect*. $N_{studies}$ defines the number of studies for each population from which data has been included for syntheses.

S4: The frequency of significant region of interest (ROI) effects for clinical samples diagnosed with MDD, PTSD, and substance use.

Percentage of significant results	MDD ($N_{studies}$ = 6)	PTSD ($N_{studies}$ = 6)	Substance Use ($N_{studies}$ = 9)
100%			
76–99%	**behavioural effect** [$N_{theoretical}$ = 6; N_{signf} = 5; 21,22–25]	**behavioural effect** [$N_{theoretical}$ = 5; N_{signf} = 4; 26,30–32]	
51–75%	**condition effect** [$N_{theoretical}$ = 6; N_{signf} = 4; 22,23–25]		**behavioural effect** [$N_{theoretical}$ = 8; N_{signf} = 5; 33,35,37,40,41]
26–50%	**training effect** [$N_{theoretical}$ = 6; N_{signf} = 3; 20,22,25], **group effect** [$N_{theoretical}$ = 6; N_{signf} = 2; 24,25], **brain–behavioural association** [$N_{theoretical}$ = 6; N_{signf} = 3; 22,24,25]	**condition effect** [$N_{theoretical}$ = 6; N_{signf} = 3; 27,29,30]	**condition effect** [$N_{theoretical}$ = 11; N_{signf} = 5; 33,34,35,39,40], **transfer effect** [$N_{theoretical}$ = 3; N_{signf} = 1; 41]
1–25%	**transfer effect** [$N_{theoretical}$ = 4; N_{signf} = 1; 25]	**training effect** [$N_{theoretical}$ = 6; N_{signf} = 1; 29], **transfer effect** [$N_{theoretical}$ = 5; N_{signf} = 1; 29], **brain–behavioural association** [$N_{theoretical}$ = 6; N_{signf} = 1; 32]	**training effect** [$N_{theoretical}$ = 11; N_{signf} = 2; 37,40], **group effect** [$N_{theoretical}$ = 6; N_{signf} = 1; 36], **brain–behavioural association** [$N_{theoretical}$ = 8; N_{signf} = 1; 35]
0%		**group effect** ($N_{theoretical}$ =5; N_{signf} = 0)	

Supplementary table 4. The frequency of significant region of interest (ROI) effects for clinical samples diagnosed with MDD, PTSD, and substance use, respectively, for the *condition effect*, *training effect*, *group effect*, *transfer effect*, *behavioural effect*, and *brain–behavioural association*. For each contrast the number of results theoretically available based on the study design ($N_{theoretical}$) and the number of significant results (N_{signf}) are presented in brackets, followed by the references of the studies that reported those significant results. Results for *brain–behavioural association* was not reported for PTSD and substance use samples and *transfer effect* was not reported for any of the three groups as less than three results were available for each respective contrast. $N_{studies}$ defines the number of studies for each population from which data has been included for syntheses. Abbreviations: MDD = Major Depressive Disorder; PTSD = Post-traumatic Stress Disorder.

S5: The frequency of significant effects for the rt-fMRI-NFB training regions of interest (ROIs) "amygdala", "PFC", "individualized multi-region ROIs", and "other ROIs"

Percentage of significant results	Amygdala ($N_{studies}$ = 13)	PFC ($N_{studies}$ = 4)	Individualized multi-region ROIs ($N_{studies}$ = 9)	Other ROIs ($N_{studies}$ = 13)
100%				
76–99%	**group effect** [$N_{theoretical}$ = 9; N_{signf} = 7; 5,6,10,17,19,24,25]			
51–75%	**training effect** [$N_{theoretical}$ = 13; N_{signf} = 8; 1,5,6,10,17,19,25,29]<br>**transfer effect** [$N_{theoretical}$ = 9; N_{signf} = 6; 5,11,13,19,25,29]		**behavioural effect** [$N_{theoretical}$ = 9; N_{signf} = 5; 22,23,35,38,40]	**behavioural effect** [$N_{theoretical}$ = 11; N_{signf} = 8; 2,3,16,26,30,31,33,41]
26–50%	**condition effect** [$N_{theoretical}$ = 13; N_{signf} = 6; 6,14,24,25,27,29]<br>**brain–behavioural association** [$N_{theoretical}$ = 9; N_{signf} = 4; 11,19,24,25]		**condition effect** [$N_{theoretical}$ = 12; N_{signf} = 5; 9,22,23,35,40]<br>**training effect** [$N_{theoretical}$ = 12; N_{signf} = 6; 8,9,20,22,37,40]	**condition effect** [3,4,18,30,$N_{theoretical}$ = 17; N_{signf} = 8; 33,34,2x 39]<br>**training effect** [$N_{theoretical}$ = 17; N_{signf} = 5; 2,3,4,16,18]<br>**brain–behavioural association** [$N_{theoretical}$ = 11; N_{signf} = 3; 2,3,16]
1–25%	**behavioural effect** [$N_{theoretical}$ = 8; N_{signf} = 2; 24,25]	**condition effect** [$N_{theoretical}$ = 6; N_{signf} = 1; 12]	**group effect** [$N_{theoretical}$ = 7; N_{signf} = 1; 8],<br>**brain–behavioural association** [$N_{theoretical}$ = 9; N_{signf} = 2; 22,35]	**group effect** [$N_{theoretical}$ = 11; N_{signf} = 2; 4,36],<br>**transfer effect** [$N_{theoretical}$ = 12; N_{signf} = 1; 41]
0%		**training effect** ($N_{theoretical}$ = 6; N_{signf} = 0),<br>**group effect** ($N_{theoretical}$ = 4; N_{signf} = 0)		

Supplementary table 5. The frequency of significant effects for the rt-fMRI-NFB training regions of interest (ROIs) "amygdala", "PFC", "individualized multi-region ROIs", and "other ROIs". For each contrast the number of results theoretically available based on the study design ($N_{theoretical}$) and the number of significant results (N_{signf}) are presented in brackets, followed by the references of the studies that reported those significant results. Studies with multiple rt-fMRI-NFB paradigms, or both clinical and healthy samples, were considered separately for *condition effects*, *training effects*, and *transfer effects*. Due to lack of sufficient data, the *training effect*, *behavioural effect*, and *brain–behavioural association* are not reported for the PFC ROI. The *transfer effect* was not reported for PFC and protocols using individualized multi-region ROIs. The following brain regions were included in the category of "other ROI": ACC (N = 5), anterior insula (N = 2), hippocampus (N = 1), orbitofrontal cortex (N = 1), PCC (N = 2), reward-related areas (N = 2). Individualized multi-region ROIs included either the amygdala, PFC,

or those regions listed under the “other ROI” category. $N_{studies}$ defines the number of studies for each population from which data has been included for syntheses. Abbreviations: NFB = neurofeedback; PFC = prefrontal cortex, ROI = region of Interest.

S6: The frequency of significant whole-brain effects for all samples, healthy samples, and clinical samples

Percentage of significant results	All samples ($N_{studies}$ = 25)	Healthy samples ($N_{studies}$ = 18)	Clinical samples ($N_{studies}$ = 13)
100%			
76–99%			
51–75%	**condition effect** [Ntheoretical = 32; N_{signf} = 17; 3,5,7,8,12,13,22,23,25,2x 30,2x 31,2x 32,2x 39]<br>**behavioural effect** [$N_{theoretical}$ = 21; N_{signf} = 12; 2,3,7,21–23,25,30–32,38,40]	**condition effect** [$N_{theoretical}$ = 18; N_{signf} = 10; 3,5,7,8,12,13,30–32,39]	**group effect** [$N_{theoretical}$ = 11; N_{signf} = 6; 21,23,25,30,31,34]<br>**behavioural effect** [$N_{theoretical}$ = 12; N_{signf} = 9; 21,22,23,25,30–32,38,40]
26–50%	**training effect** [$N_{theoretical}$ = 32; N_{signf} = 16; 2,6,8,9,12,17,2x 21,22,28,2x 31,34,2x 37,38],<br>**group effect** [$N_{theoretical}$ = 19; N_{signf} = 8; 6,17,21,23,25,30,31,34]	**training effect** [$N_{theoretical}$ = 18; N_{signf} = 9; 2,6,8,9,12,17,21,31,37],<br>**transfer effect** [$N_{theoretical}$ = 9; N_{signf} = 3; 13,19,31]<br>**behavioural effect** [$N_{theoretical}$ = 9; N_{signf} = 3; 2,3,7]	**condition effect** [$N_{theoretical}$ = 14; N_{signf} = 7; 22,23,25,30–32,39],<br>**training effect** [$N_{theoretical}$ = 14; N_{signf} = 7; 21,22,28,31,34,37,38]
1–25%	**transfer effect** [$N_{theoretical}$ = 16; N_{signf} = 4; 13,19,2x 31]	**group effect** [$N_{theoretical}$ = 8; N_{signf} = 2; 6,17]	**transfer effect** [$N_{theoretical}$ = 7; N_{signf} = 1; 31]
0%			

Supplementary table 6. The frequency of significant whole-brain effects for all samples, healthy samples, and clinical samples for *condition effect*, *training effect*, *group effect*, *transfer effect*, *behavioural effect*, and *brain–behavioural association*. For each contrast the number of results theoretically available based on the study design ($N_{theoretical}$) and the number of significant results (N_{signf}) are presented in brackets, followed by the references of the studies that reported those significant results. As some studies included both healthy and clinical samples, results for both samples have been considered for calculation of *condition effect*, *training effect* and *transfer effect*. Results for *transfer effect* and *brain–behavioural association* could not be calculated due to lack of at least three available results for these contrasts. $N_{studies}$ defines the number of studies for each population from which data has been included for syntheses.

References

1. Bruhl, A.; Scherpiet, S.; Sulzer, J.; Stampfli, P.; Seifritz, E.; Herwig, U. Real-time Neurofeedback Using Functional MRI Could Improve Down-Regulation of Amygdala Activity During Emotional Stimulation: A Proof-of-Concept Study. *Brain Topography* **2014**, *27*, 138-148, doi:10.1007/s10548-013-0331-9.
2. Caria, A.; Sitaram, R.; Veit, R.; Begliomini, C.; Birbaumer, N. Volitional Control of Anterior Insula Activity Modulates the Response to Aversive Stimuli. A Real-Time Functional Magnetic Resonance Imaging Study. *Biological Psychiatry* **2010**, *68*, 425-432, doi:10.1016/j.biopsych.2010.04.020.
3. Gröne, M.; Dyck, M.; Koush, Y.; Bergert, S.; Mathiak, K.A.; Alawi, E.M.; Elliott, M.; Mathiak, K. Upregulation of the Rostral Anterior Cingulate Cortex can Alter the Perception of Emotions: fMRI-Based Neurofeedback at 3 and 7 T. *Brain Topography* **2015**, *28*, 197-207, doi:10.1007/s10548-014-0384-4.
4. Hamilton, J.P.; Glover, G.H.; Hsu, J.J.; Johnson, R.F.; Gotlib, I.H. Modulation of subgenual anterior cingulate cortex activity with real-time neurofeedback. *Hum Brain Mapp* **2011**, *32*, 22-31, doi:10.1002/hbm.20997.
5. Hellrung, L.; Dietrich, A.; Hollmann, M.; Pleger, B.; Kalberlah, C.; Roggenhofer, E.; Villringer, A.; Horstmann, A. Intermittent compared to continuous real-time fMRI neurofeedback boosts control over amygdala activation. *Neuroimage* **2018**, *166*, 198-208, doi:10.1016/j.neuroimage.2017.10.031.
6. Herwig, U.; Lutz, J.; Scherpiet, S.; Scheerer, H.; Kohlberg, J.; Opialla, S.; Preuss, A.; Steiger, V.R.; Sulzer, J.; Weidt, S.; et al. Training emotion regulation through real-time fMRI neurofeedback of amygdala activity. *Neuroimage* **2019**, *184*, 687-696, doi:10.1016/j.neuroimage.2018.09.068.
7. Ihssen, N.; Sokunbi, M.O.; Lawrence, A.D.; Lawrence, N.S.; Linden, D.E.J. Neurofeedback of visual food cue reactivity: a potential avenue to alter incentive sensitization and craving. *Brain Imaging and Behavior* **2017**, *11*, 915-924, doi:10.1007/s11682-016-9558-x.
8. Johnston, S.; Linden, D.E.; Healy, D.; Goebel, R.; Habes, I.; Boehm, S.G. Upregulation of emotion areas through neurofeedback with a focus on positive mood. *Cogn Affect Behav Neurosci* **2011**, *11*, 44-51, doi:10.3758/s13415-010-0010-1.
9. Johnston, S.J.; Boehm, S.G.; Healy, D.; Goebel, R.; Linden, D.E. Neurofeedback: A promising tool for the self-regulation of emotion networks. *Neuroimage* **2010**, *49*, 1066-1072, doi:10.1016/j.neuroimage.2009.07.056.
10. Liu, N.; Yao, L.; Zhao, X. Evaluating the amygdala network induced by neurofeedback training for emotion regulation using hierarchical clustering. *Brain Research* **2020**, *1740*, doi:10.1016/j.brainres.2020.146853.
11. Marxen, M.; Jacob, M.J.; Muller, D.K.; Posse, S.; Ackley, E.; Hellrung, L.; Riedel, P.; Bender, S.; Epple, R.; Smolka, M.N. Amygdala regulation following fMRI-neurofeedback without instructed strategies. *Frontiers in Human Neuroscience* **2016**, *10(APR2016)*, 1-14, doi:https://dx.doi.org/10.3389/fnhum.2016.00183.
12. Mayeli, A.; Misaki, M.; Zotev, V.; Tsuchiyagaito, A.; Al Zoubi, O.; Phillips, R.; Smith, J.; Stewart, J.L.; Refai, H.; Paulus, M.P.; et al. Self-regulation of ventromedial prefrontal cortex activation using real-time fMRI neurofeedback-Influence of default mode network. *Human brain mapping.* **2019**, *21*, doi:https://dx.doi.org/10.1002/hbm.24805.
13. Paret, C.; Kluetsch, R.; Ruf, M.; Demirakca, T.; Hoesterey, S.; Ende, G.; Schmahl, C. Down-Regulation of amygdala activation with Real-Time fmri neurofeedback in a healthy female sample. *Frontiers in Behavioral Neuroscience* **2014**, *8 (no pagination)*, doi:https://dx.doi.org/10.3389/fnbeh.2014.00299.
14. Paret, C.; Zahringer, J.; Ruf, M.; Gerchen, M.F.; Mall, S.; Hendler, T.; Schmahl, C.; Ende, G. Monitoring and control of amygdala neurofeedback involves distributed information processing in the human brain. *Human Brain Mapping* **2018**, *39(7)*, 3018-3031, doi:https://dx.doi.org/10.1002/hbm.24057.

15. Sarkheil, P.; Zilverstand, A.; Kilian-Hutten, N.; Schneider, F.; Goebel, R.; Mathiak, K. fMRI feedback enhances emotion regulation as evidenced by a reduced amygdala response. *Behavioural Brain Research* **2015**, *281*, 326-332, doi:https://dx.doi.org/10.1016/j.bbr.2014.11.027.
16. Scheinost, D.; Stoica, T.; Saksa, J.; Papademetris, X.; Constable, R.T.; Pittenger, C.; Hampson, M. Orbitofrontal cortex neurofeedback produces lasting changes in contamination anxiety and resting-state connectivity. *Translational Psychiatry* **2013**, *3 (no pagination)*, doi:https://dx.doi.org/10.1038/tp.2013.24.
17. Wang, Y.; Yao, L.; Zhao, X. Amygdala network in response to facial expression following neurofeedback training of emotion. *Brain imaging and behavior* **2020**, *14(3)*, 897-906, doi:https://dx.doi.org/10.1007/s11682-019-00052-4.
18. Zhu, Y.; Gao, H.; Tong, L.; Li, Z.; Wang, L.; Zhang, C.; Yang, Q.; Yan, B. Emotion regulation of hippocampus using real-time fMRI neurofeedback in healthy human. *Frontiers in human neuroscience* **2019**, *13*, doi:10.3389/fnhum.2019.00242.
19. Zotev, V.; Krueger, F.; Phillips, R.; Alvarez, R.P.; Simmons, W.K.; Bellgowan, P.; Drevets, W.C.; Bodurka, J. Self-regulation of amygdala activation using real-time FMRI neurofeedback. *PloS one* **2011**, *6*, e24522, doi:10.1371/journal.pone.0024522.
20. Hamilton, J.P.; Glover, G.H.; Bagarinao, E.; Chang, C.; Mackey, S.; Sacchet, M.D.; Gotlib, I.H. Effects of salience-network-node neurofeedback training on affective biases in major depressive disorder. *Psychiatry Research-Neuroimaging* **2016**, *249*, 91-96, doi:10.1016/j.pscychresns.2016.01.016.
21. Keller, M.; Zweerings, J.; Klasen, M.; Zvyagintsev, M.; Iglesias, J.; Mendoza Quiñones, R.; Mathiak, K. fMRI Neurofeedback-Enhanced Cognitive Reappraisal Training in Depression: A Double-Blind Comparison of Left and Right vlPFC Regulation. *Front Psychiatry* **2021**, *12*, 715898, doi:10.3389/fpsyt.2021.715898.
22. Linden, D.E.; Habes, I.; Johnston, S.J.; Linden, S.; Tatineni, R.; Subramanian, L.; Sorger, B.; Healy, D.; Goebel, R. Real-time self-regulation of emotion networks in patients with depression. *PLoS One* **2012**, *7*, e38115, doi:10.1371/journal.pone.0038115.
23. Mehler, D.M.A.; Sokunbi, M.O.; Habes, I.; Barawi, K.; Subramanian, L.; Range, M.; Evans, J.; Hood, K.; Luhrs, M.; Keedwell, P.; et al. Targeting the affective brain—a randomized controlled trial of real-time fMRI neurofeedback in patients with depression. *Neuropsychopharmacology* **2018**, *43*, 2578-2585, doi:10.1038/s41386-018-0126-5.
24. Young, K.D.; Siegle, G.J.; Zotev, V.; Phillips, R.; Misaki, M.; Yuan, H.; Drevets, W.C.; Bodurka, J. Randomized clinical trial of real-time fMRI amygdala neurofeedback for major depressive disorder: effects on symptoms and autobiographical memory recall. *American Journal of Psychiatry* **2017**, *174*, 748-755.
25. Young, K.D.; Zotev, V.; Phillips, R.; Misaki, M.; Yuan, H.; Drevets, W.C.; Bodurka, J. Real-time fMRI neurofeedback training of amygdala activity in patients with major depressive disorder. *PLoS ONE* **2014**, *9*, doi:10.1371/journal.pone.0088785.
26. Lieberman, J.M.; Rabellino, D.; Densmore, M.; Frewen, P.A.; Steyrl, D.; Scharnowski, F.; Theberge, J.; Neufeld, R.W.J.; Schmahl, C.; Jetly, R.; et al. Posterior cingulate cortex targeted real-time fMRI neurofeedback recalibrates functional connectivity with the amygdala, posterior insula, and default-mode network in PTSD. *Brain and Behavior* **2023**, *13*, doi:10.1002/brb3.2883.
27. Misaki, M.; Phillips, R.; Zotev, V.; Wong, C.K.; Wurfel, B.E.; Krueger, F.; Feldner, M.; Bodurka, J. Brain activity mediators of PTSD symptom reduction during real-time fMRI amygdala neurofeedback emotional training. *NeuroImage: Clinical* **2019**, *24 (no pagination)*, doi:https://dx.doi.org/10.1016/j.nicl.2019.102047.
28. Nicholson, A.A.; Rabellino, D.; Densmore, M.; Frewen, P.A.; Paret, C.; Kluetsch, R.; Schmahl, C.; Théberge, J.; Neufeld, R.W.; McKinnon, M.C.; et al. The neurobiology of emotion regulation in posttraumatic stress disorder: Amygdala downregulation via real-time fMRI neurofeedback. *Hum Brain Mapp* **2017**, *38*, 541-560, doi:10.1002/hbm.23402.

29. Nicholson, A.A.; Rabellino, D.; Densmore, M.; Frewen, P.A.; Paret, C.; Kluetsch, R.; Schmahl, C.; Theberge, J.; Ros, T.; Neufeld, R.W.J.; et al. Intrinsic connectivity network dynamics in PTSD during amygdala downregulation using real-time fMRI neurofeedback: A preliminary analysis. *Human Brain Mapping* **2018**, *39(11)*, 4258-4275, doi:https://dx.doi.org/10.1002/hbm.24244.
30. Nicholson, A.A.; Rabellino, D.; Densmore, M.; Frewen, P.A.; Steryl, D.; Scharnowski, F.; Theberge, J.; Neufeld, R.W.J.; Schmahl, C.; Jetly, R.; et al. Differential mechanisms of posterior cingulate cortex downregulation and symptom decreases in posttraumatic stress disorder and healthy individuals using real-time fMRI neurofeedback. *Brain and Behavior* **2022**, *12(1) (no pagination)*, doi:https://dx.doi.org/10.1002/brb3.2441.
31. Zweerings, J.; Pflieger, E.M.; Mathiak, K.A.; Zvyagintsev, M.; Kacela, A.; Flatten, G.; Mathiak, K. Impaired voluntary control in PTSD: Probing self-regulation of the ACC with real-time fMRI. *Frontiers in Psychiatry* **2018**, *9(MAY) (no pagination)*, doi:https://dx.doi.org/10.3389/fpsyt.2018.00219.
32. Zweerings, J.; Sarkheil, P.; Keller, M.; Dyck, M.; Klasen, M.; Becker, B.; Gaebler, A.J.; Ibrahim, C.N.; Turetsky, B.I.; Zvyagintsev, M.; et al. Rt-fMRI neurofeedback-guided cognitive reappraisal training modulates amygdala responsivity in posttraumatic stress disorder. *NeuroImage. Clinical* **2020**, *28*, 102483, doi:10.1016/j.nicl.2020.102483.
33. Canterberry, M.; Hanlon, C.A.; Hartwell, K.J.; Li, X.; Owens, M.; LeMatty, T.; Prisciandaro, J.J.; Borckardt, J.; Saladin, M.E.; Brady, K.T. Sustained reduction of nicotine craving with real-time neurofeedback: exploring the role of severity of dependence. *Nicotine & tobacco research* **2013**, *15*, 2120-2124.
34. Chung, Y.I.; White, R.; Geier, C.F.; Johnston, S.J.; Smyth, J.M.; Delgado, M.R.; McKee, S.A.; Wilson, S.J. Testing the efficacy of real-time fMRI neurofeedback for training people who smoke daily to upregulate neural responses to nondrug rewards. *Cognitive Affective & Behavioral Neuroscience* **2023**, *23*, 440-456, doi:10.3758/s13415-023-01070-y.
35. Hanlon, C.A.; Hartwell, K.J.; Canterberry, M.; Li, X.; Owens, M.; LeMatty, T.; Prisciandaro, J.J.; Borckardt, J.; Brady, K.T.; George, M.S. Reduction of cue-induced craving through realtime neurofeedback in nicotine users: the role of region of interest selection and multiple visits. *Psychiatry Research: Neuroimaging* **2013**, *213*, 79-81.
36. Hartwell, K.J.; Hanlon, C.A.; Li, X.; Borckardt, J.J.; Canterberry, M.; Prisciandaro, J.J.; Moran-Santa Maria, M.M.; LeMatty, T.; George, M.S.; Brady, K.T. Individualized real-time fMRI neurofeedback to attenuate craving in nicotine-dependent smokers. *Journal of Psychiatry and Neuroscience* **2016**, *41*, 48-55.
37. Karch, S.; Keeser, D.; Hümmer, S.; Paolini, M.; Kirsch, V.; Karali, T.; Kupka, M.; Rauchmann, B.-S.; Chrobok, A.; Blautzik, J. Modulation of craving related brain responses using real-time fMRI in patients with alcohol use disorder. *PLoS One* **2015**, *10*, e0133034.
38. Karch, S.; Krause, D.; Lehnert, K.; Konrad, J.; Haller, D.; Rauchmann, B.-S.; Maywald, M.; Engelbregt, H.; Adorjan, K.; Koller, G. Functional and clinical outcomes of FMRI-based neurofeedback training in patients with alcohol dependence: a pilot study. *European archives of psychiatry and clinical neuroscience* **2021**, 1-13.
39. Kirschner, M.; Sladky, R.; Haugg, A.; Stampfli, P.; Jehli, E.; Hodel, M.; Engeli, E.; Hosli, S.; Baumgartner, M.R.; Sulzer, J.; et al. Self-regulation of the dopaminergic reward circuit in cocaine users with mental imagery and neurofeedback. *EBioMedicine* **2018**, *37*, 489-498, doi:https://dx.doi.org/10.1016/j.ebiom.2018.10.052.
40. Li, X.; Hartwell, K.J.; Borckardt, J.; Prisciandaro, J.J.; Saladin, M.E.; Morgan, P.S.; Johnson, K.A.; LeMatty, T.; Brady, K.T.; George, M.S. Volitional reduction of anterior cingulate cortex activity produces decreased cue craving in smoking cessation: A preliminary real-time fMRI study. *Addiction biology* **2013**, *18*, 739-748.
41. Rana, M.; Ruiz, S.; Corzo, A.S.; Muehleck, A.; Eck, S.; Salinas, C.; Zamorano, F.; Silva, C.; Rea, M.; Batra, A. Use of real-time functional magnetic resonance imaging-based neurofeedback to downregulate insular cortex in nicotine-addicted smokers. *J vis Exp* **2020**, *160*, e59441.
